# Glucocorticoid receptor alters isovolumetric contraction and restrains cardiac fibrosis

**DOI:** 10.1530/JOE-16-0458

**Published:** 2017-01-05

**Authors:** Rachel V Richardson, Emma J Batchen, Adrian J W Thomson, Rowan Darroch, Xinlu Pan, Eva A Rog-Zielinska, Wiktoria Wyrzykowska, Kathleen Scullion, Emad A S Al-Dujaili, Mary E Diaz, Carmel M Moran, Christopher J Kenyon, Gillian A Gray, Karen E Chapman

**Affiliations:** 1 University/BHF Centre for Cardiovascular ScienceUniversity of Edinburgh, The Queen’s Medical Research Institute, Edinburgh, UK; 2 Edinburgh Preclinical ImagingUniversity of Edinburgh, Edinburgh, UK; 3 DieteticsNutrition, and Biological Sciences Department, Queen Margaret University, Musselburgh, UK

**Keywords:** glucocorticoid receptor, mineralocorticoid receptor, heart, fibrosis, isovolumetric contraction

## Abstract

Corticosteroids directly affect the heart and vasculature and are implicated in the pathogenesis of heart failure. Attention is focussed upon the role of the mineralocorticoid receptor (MR) in mediating pro-fibrotic and other adverse effects of corticosteroids upon the heart. In contrast, the role of the glucocorticoid receptor (GR) in the heart and vasculature is less well understood. We addressed this in mice with cardiomyocyte and vascular smooth muscle deletion of GR (SMGRKO mice). Survival of SMGRKO mice to weaning was reduced compared with that of littermate controls. Doppler measurements of blood flow across the mitral valve showed an elongated isovolumetric contraction time in surviving adult SMGRKO mice, indicating impairment of the initial left ventricular contractile phase. Although heart weight was elevated in both genders, only male SMGRKO mice showed evidence of pathological cardiomyocyte hypertrophy, associated with increased myosin heavy chain-β expression. Left ventricular fibrosis, evident in both genders, was associated with elevated levels of mRNA encoding MR as well as proteins involved in cardiac remodelling and fibrosis. However, MR antagonism with spironolactone from birth only modestly attenuated the increase in pro-fibrotic gene expression in SMGRKO mice, suggesting that elevated MR signalling is not the primary driver of cardiac fibrosis in SMGRKO mice, and cardiac fibrosis can be dissociated from MR activation. Thus, GR contributes to systolic function and restrains normal cardiac growth, the latter through gender-specific mechanisms. Our findings suggest the GR:MR balance is critical in corticosteroid signalling in specific cardiac cell types.

## Introduction

Corticosteroids, mineralocorticoids and glucocorticoids, exert a variety of effects on the cardiovascular system. Excessive glucocorticoid levels, whether endogenous as in Cushing’s disease, or exogenous through pharmacotherapy, are associated with increased risk of cardiovascular disease and heart failure (Souverein *et al*. 2004, Walker 2007). In contrast, physiological levels of glucocorticoids are important in the homeostatic regulation of the heart and vasculature (Richardson *et al*. 2016). In Addison’s disease, insufficient glucocorticoid action leads to myocardial weakness and reduced cardiac output, though the underlying reasons are complex and remain poorly understood. In mice, long-term adrenalectomy induces cardiac abnormalities that include enlarged hearts and impaired left ventricular (LV) function, both of which are rescued by glucocorticoid (corticosterone) replacement therapy (Cruz-Topete *et al*. 2016).

Endogenous glucocorticoids exert most of their actions through the closely related glucocorticoid receptor (GR) and mineralocorticoid receptor (MR). Cardiomyocytes lack the glucocorticoid-inactivating enzyme, 11β-hydroxysteroid dehydrogenase type 2 (Chapman *et al*. 2013). Thus, the effects of glucocorticoids in cardiomyocytes are potentially mediated by both GR and MR. Both receptors are capable of binding to and activating many of the same target genes. Nevertheless, they play distinct, and sometimes opposing, roles in glucocorticoid action (Richardson *et al*. 2016). Elucidation of their respective physiological roles in the cardiovascular system is important to understand their pathophysiological relevance and may suggest new therapeutic approaches to cardiac disease.

Elevated MR signalling is pro-inflammatory in the heart and associates with cardiac fibrosis, inflammation and heart failure (Messaoudi *et al*. 2012, Young & Rickard 2015). The effectiveness of MR antagonism in the treatment of heart failure has focussed its attention on the significance of MR activation in heart. However, deletion of MR in cardiomyocytes has a relatively minor impact on the heart in normal physiology (Fraccarollo *et al*. 2011, Lother *et al*. 2011). Similarly, although vascular smooth muscle (VSM) MR knockout is protective against age-related hypertension and cardiac hypertrophy, it has negligible effect on blood pressure and heart size in young mice (<7 months of age) (McCurley *et al*. 2012). The physiological role of GR in the cardiovascular system has been less explored. We have previously shown an essential role for GR in maturing the foetal heart in preparation for life after birth. Hearts of foetal SMGRKO mice, which lack GR in cardiomyocytes and VSM, showed evidence of functional, structural and biochemical immaturity (Rog-Zielinska *et al*. 2013). Consistent with this, glucocorticoid treatment of primary murine foetal cardiomyocytes, improved contractility, calcium handling and myofibril structure in a GR-dependent manner (Rog-Zielinska *et al*. 2014*a*). Others have started to address the role of GR signalling in adult heart. Recent evidence in mice with *MHCα-Cre*-mediated deletion of GR in cardiomyocytes has demonstrated a tonic role for GR in preventing heart disease, with anti-inflammatory and pro-survival/anti-apoptotic mechanisms implicated (Oakley *et al*. 2013).

Here, we aimed to establish the postnatal consequences of *SM22α-Cre*-driven deletion of GR in cardiomyocytes and VSM for cardiac function and remodelling using SMGRKO mice. We show that the isovolumetric contraction time is increased in adult male and female SMGRKO mice, demonstrating a homeostatic role for GR in normal systolic function in both genders. Heart mass is increased in adult mice of both genders, yet cardiomyocyte hypertrophy only develops in male, and not female SMGRKO mice, suggesting gender-specific roles of glucocorticoids in the development of cardiomyocyte hypertrophy. In both genders, mice show evidence of cardiac fibrosis that is associated with increased expression of MR. However, MR antagonism only partially rescues the pro-fibrotic phenotype indicating that MR is not the primary driver of cardiac fibrosis in this model. SMGRKO mice may be a useful pre-clinical model in which to investigate MR-independent pathways that drive cardiac fibrosis.

## Materials and methods

### Animals

All experiments involving animals were approved by the University of Edinburgh Animal Welfare and Ethical Review Body and were carried out in strict accord with accepted standards of humane animal care under the auspices of the Animal (Scientific Procedures) Act UK 1986. SMGRKO mice were generated by crossing mice with *loxP* sites flanking exon 3 of the *Nr3c1* gene encoding GR (GR^fl/fl^ mice), congenic on a C57BL/6J genetic background, with *SM22α-Cre* transgenic mice as described previously (Rog-Zielinska *et al*. 2013). In all experiments, *Cre^+^* SMGRKO mice were compared with *Cre^−^* (control) littermates. Cardiac function was assessed at 10 weeks of age using a Visualsonics Vevo 770 High Resolution Ultrasound Scanner. Briefly, mice were anaesthetised with 2% isoflurane gas, and the parasternal long axis view was used to image the heart in M-mode and in ECG-Gated Kilohertz Visualization (EKV) mode, whilst body temperature was maintained at 37°C and heart rate was maintained at ~450 bpm. Pulse-wave Doppler was used to measure blood flow across the mitral valve chamber using the apical four chamber view. Measurements were collected from the Doppler traces using Vevo 770 image analysis software. Mice were decapitated at 12 weeks of age and trunk blood was collected. Kidneys were weighed, snap frozen and stored at −80°C for subsequent RNA and protein analysis. Hearts from male mice were weighed then either fixed in 10% formalin for histological and immunohistochemical analysis or were snap frozen and stored at −80°C for subsequent RNA and protein analysis. Hearts from female mice were weighed and a 5 mm transverse segment of heart was fixed in 10% formalin. The remaining portions of the LV were snap frozen for mRNA and protein extraction. For retrograde cardiac perfusion-fixation, male mice were anaesthetised by intraperitoneal injection of 1 mg/kg medetomidine with 75 mg/kg ketamine. The abdominal aorta was cannulated below the branch of the renal artery and perfused with heparinised PBS for 2 min followed by 10% formalin in PBS for 5 min. Hearts were dissected and fixed in 10% formalin.

### Spironolactone treatment

Male SMGRKO mice and control littermates were treated from birth with vehicle or 20 mg/kg/day spironolactone, an MR antagonist, administered in the drinking water to lactating dams until weaning then to offspring, according to a previous report (Ouvrard-Pascaud *et al*. 2005). Animals were killed by cervical dislocation at 8 weeks for organ collection. Tissues were collected as mentioned previously.

### Ventricular cardiomyocyte isolation and volume measurement

Cardiomyocytes were isolated following retrograde Langendorff perfusion with collagenase type 1 (0.6 mg/mL, Worthington, New Jersey, USA) and protease type XIV (0.075 mg/mL, Sigma-Aldrich) in a modified Tyrode’s solution containing 134 mM NaCl, 11 mM Glucose, 4 mM KCl, 1.2 mM MgSO_4_, 1.2 mM Na_2_H_2_PO_4_, 10 mM HEPES, pH 7.34. Rod-shaped cardiomyocytes were collected in the same modified Tyrode’s solution but containing 108 mM NaCl with the addition of 50 mM taurine and 50 µM CaCl_2_. Cardiomyocyte volume was estimated from the cell membrane surface area (Page 1978), calculated by applying a hyperpolarizing pulse (−10 mV) under voltage-clamp and integrating the resulting capacitive current, as described (Diaz *et al*. 1997).

### Plasma corticosterone measurements

Plasma was obtained from blood samples collected from the tail vein of conscious mice at 07:00 h and 19:00 h and stored at −80°C prior to use. Corticosterone levels were measured using a colourimetric ELISA as described (Al-Dujaili *et al*. 2009).

### Western blotting

Protein was extracted from left ventricle (LV) tissue by homogenising in protein extraction buffer (25 mM HEPES, 68.5 mM NaCl, 0.5 mM MgCl_2_, 0.5 mM CaCl_2_, 5 mM NaF, 1 mM EDTA, 5 mM sodium pyrophosphate, 1% NP-40, 10% glycerol, 1× protease inhibitor cocktail tablet). After centrifugation, protein concentration was measured relative to bovine serum albumin (Protein assay standard II, Bio-Rad) using Bradford assay. Protein samples (30 μg) were added to NuPAGE LDS Sample Buffer and NUPAGE Sample Reducing Buffer (both Thermo Fisher Scientific). Samples were denatured, electrophoresed on a NuPAGE Novex 4–12% Bis Tris gel in NuPAGE MES SDS running buffer (Thermo Fisher Scientific), and then transferred to a nitrocellulose membrane. The membrane was blocked with 5% BSA in Tris-buffered saline for 1 h, and then incubated with primary antibodies against GR (1:400; M-20, Insight Biotechnology, Middlesex, UK) and β-actin (1:10,000; 4967S Cell Signalling Technologies) at 4°C overnight, washed and incubated with secondary antibodies: IRDye 800CW Goat anti-Rabbit IgG (1:10,000; 926-32211 Licor Biosciences, Cambridge, UK) and IRDye 800CW Goat anti-Mouse IgG (1:10,000; 926-32210 Licor Biosciences). An Odyssey infrared imaging system (Licor Biosciences) was used to measure GR protein levels relative to β-actin using Odyssey software.

### qRT-PCR

RNA was extracted from LV tissue by homogenisation in TRIzol (Thermo Fisher Scientific) followed by purification using a Purelink RNA mini kit (Thermo Fisher Scientific). cDNA was synthesised from 1 μg RNA using a SuperScript III Reverse transcriptase system kit (Thermo Fisher Scientific) then subject to quantitative (q)PCR (in triplicate) using the Roche Lightcycler 480 system and universal probe library (Roche) with gene-specific primer sets (Supplementary Table 1, see section on supplementary data given at the end of this article). A standard curve was prepared from pooled cDNA samples. Relative quantification was provided by LightCycler software using the maximum second derivative method and mRNA levels were normalised to the average of *Actb* and *Gapdh* mRNA levels.

### Mouse fibrosis PCR array

cDNA was synthesised from 1µg mouse LV RNA using the RT^2^ First Strand Kit (Qiagen) according to the manufacturer’s instructions. cDNA was mixed with RT^2^ Syber Green Mastermix prior to addition to the Mouse Fibrosis RT^2^ Profiler PCR Array plate (PAMM-120E, Qiagen). Plates were heated to 95°C for 10 min then underwent 40 cycles of amplification (95°C, 15 s then 60°C, 1 min) using an Applied Biosystems 7900 Real-Time PCR System (Thermo Fisher Scientific). The threshold value was manually set and *C*_T_ values were exported for analysis.

### Histological and immunohistochemical analysis

Fixed hearts were embedded in paraffin and sectioned (7 μm). For quantification of fibrosis, deparaffinised and rehydrated sections were stained with Direct Red 80/Fast green in aqueous picric acid. Four fields of view were scored from the LV of each mouse by an investigator blind to genotype (0 = no interstitial collagen; 1 = subtle traces of interstitial collagen; 2 = clearly visible thin strands of interstitial collagen; 3 = thicker, more numerous strands of interstitial collagen; 4 = severe focal patches of interstitial collagen deposition in addition to thick interstitial collagen strands). To measure cardiomyocyte cross-sectional area, sections were stained with Isolectin B4 (1:100; I21414 Thermo Fisher Scientific) followed by streptavidin Alexa 488 (1:100; S32354 Thermo Fisher Scientific) in combination with rhodamine-conjugated wheat germ agglutinin (1:1000; RL-1022 Vector Laboratories, Peterborough, UK) and DAPI. Cardiomyocytes were counted and measured using Adobe Photoshop CS5. To assess cardiomyocyte proliferation in neonatal hearts, sections were stained with anti-Cardiac Troponin T (1F11) (1:200; ab10214 Abcam) in combination with anti-Ki67 (1:200; ab15580 Abcam). Flurophore-conjugated secondary antibodies (Alexa Fluor, Invitrogen) were used to detect immunostaining. The percentage of Ki67-positive cardiomyocytes was calculated using Adobe Photoshop CS5.

### Statistical analysis

Statistical analysis was performed using GraphPad Prism 6 software. The impact of genotype on the survival of mice was assessed using the Chi-square test. Comparisons between two groups were made using an unpaired *t*-test. For analysis of two factors, a two-way ANOVA was carried out with multiple comparisons made using the Tukey *post hoc* test. Values are means ± s.e.m. with *P* < 0.05 deemed significant.

## Results

### SMGRKO mice lack glucocorticoid receptor expression in cardiomyocytes and VSM and show reduced early-life survival

**Figure 1:**
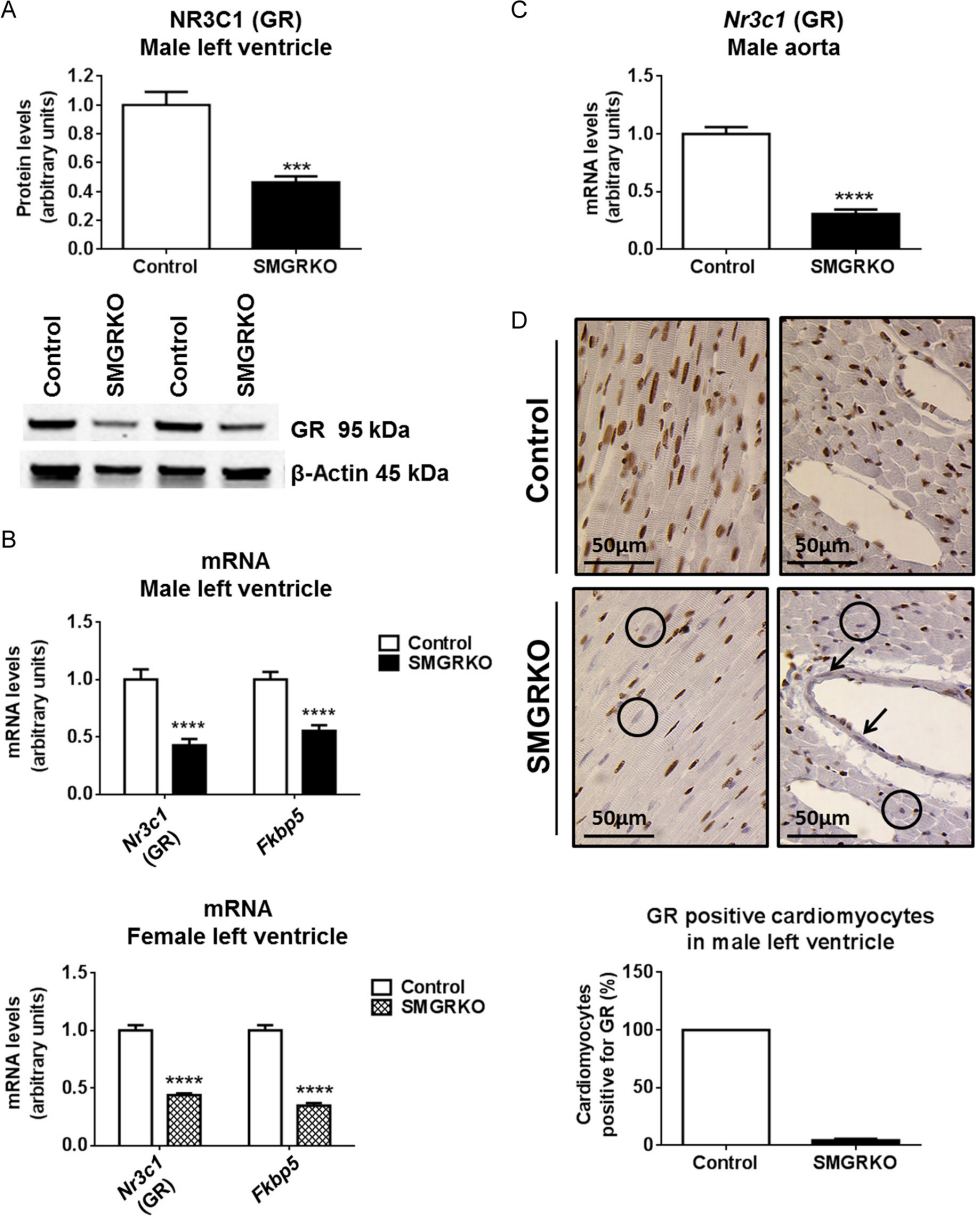
Adult SMGRKO mice lack glucocorticoid receptor expression in cardiomyocytes and vascular smooth muscle cells. Left ventricles (LV) and aorta were collected from 12-week-old SMGRKO mice (black/hatched bars) and control littermates (white bars). (A) Western blotting shows NR3C1 (GR) protein levels (relative to internal control β-actin) are reduced in LV of male SMGRKO mice (*N* = 5–6). (B) Quantitative RT-PCR shows reduced levels of mRNA encoding GR (*Nr3c1*) and FK506-binding protein 5 (*Fkbp5*) in LV of male and female SMGRKO mice, compared to controls (*N* = 9–12). (C) Levels of *Nr3c1* mRNA are reduced in aortas of male SMGRKO mice compared to those in controls (*N* = 7–9). (D) Representative images of immunohistochemical staining of GR (brown DAB staining) in LV of male SMGRKO mice. Circles indicate examples of cardiomyocytes lacking GR and arrows indicate vascular smooth muscle cells lacking GR. Cardiomyocytes cut in the short axis were scored for the presence or absence of GR staining (8 fields of view/heart) (*N* = 6–7). Data are means ± s.e.m. and were analysed using an unpaired *t*-test. ***P < 0.001, ****P < 0.0001.

The *SM22α-Cre* transgene used to produce SMGRKO mice is transiently expressed in cardiomyocytes as well as in VSM (Li *et al*. 1996). We have previously shown that GR protein and mRNA levels are reduced in whole hearts of foetal SMGRKO (*Cre+*) mice (Rog-Zielinska *et al*. 2013). Consistent with this, levels of GR protein and mRNA were reduced in hearts of adult SMGRKO mice compared with controls (Left ventricle, LV). In accordance with the known activity of *SM22α-Cre* in VSM cells, levels of *Nr3c1* mRNA (encoding GR) were also reduced in the aorta of SMGRKO mice (Fig. 1C). Immunohistochemical staining showed that the reduction in LV GR protein levels is in cardiomyocytes and VSM cells (Fig. 1D). Levels of mRNA encoding the GR target gene, FK506-binding protein-5 (*Fkbp5*), were reduced in LV of SMGRKO mice compared with those in controls (Fig. 1B), consistent with reduced GR signalling in hearts of SMGRKO mice.

Although foetal SMGRKO mice do not deviate from the expected 50% Mendelian ratio at embryonic day (E)17.5 (Rog-Zielinska *et al*. 2013), by weaning (at 3 weeks of age) SMGRKO mice were only 40% of the total male and 36% of the total female population (males: SMGRKO = 94 vs control = 142, chi-squared test *P* < 0.01; females: SMGRKO = 92 vs control = 163; chi-squared test *P* < 0.0001. This suggests that only 66% of male and 56% of female SMGRKO mice survive weaning. Very few deaths occurred following recording of litters (normally within 1 day of birth) suggesting that deaths occur in late gestation or shortly after birth. Following weaning, survival was similar between SMGRKO and control mice.

### Systolic cardiac function is impaired in adult SMGRKO mice

**Figure 2:**
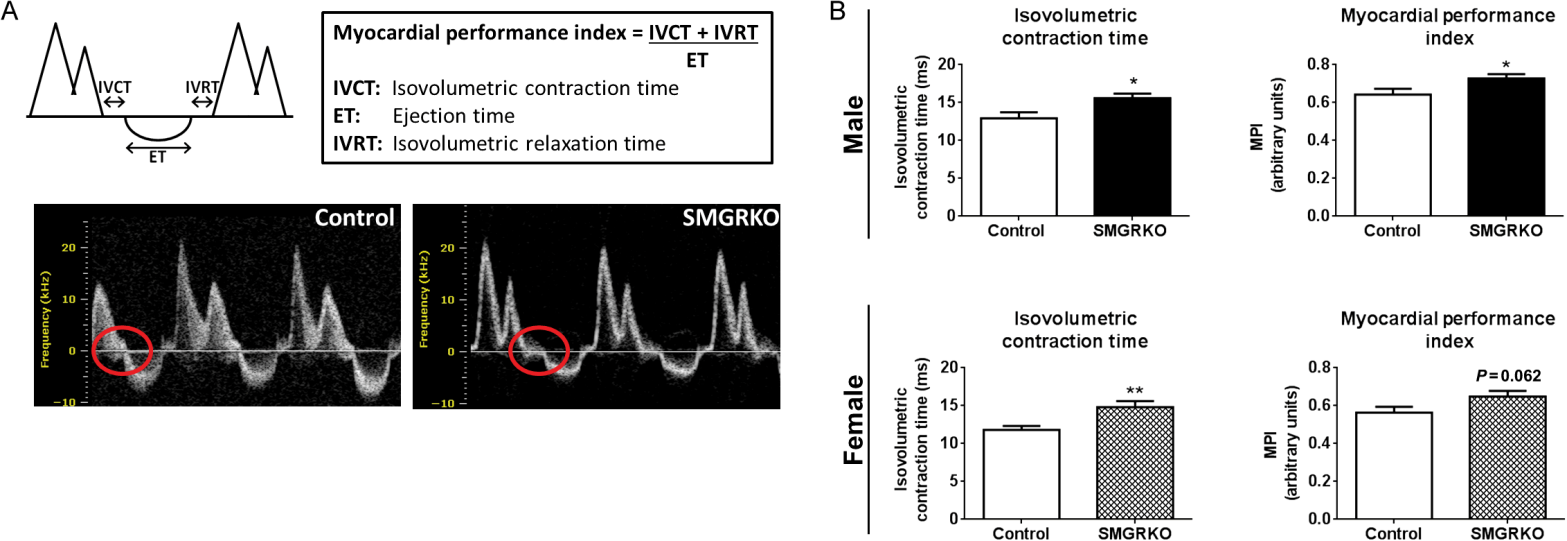
Doppler measurements of blood flow across the mitral valve within the LV show impaired cardiac function in 10-week-old SMGRKO mice. (A) Schematic representation of a Doppler trace indicating isovolumetric contraction time (IVCT), ejection time (ET) and isovolumetric relaxation time (IVRT) used to derive the myocardial performance index (MPI). Representative Doppler traces highlight the elongated IVCT in SMGRKO mice, compared to littermate controls (circled). (B) Elongation of the IVCT underlies the detrimental elevation in MPI in 10-week-old SMGRKO mice (black/hatched bars) compared to littermate controls (white bars) (*N* = 11–16). Data are means ± s.e.m. and were analysed using an unpaired *t*-test. *P < 0.05, **P < 0.01.

High-resolution ultrasound was used to assess the functional consequence of reduced GR expression in cardiomyocytes and VSM in adult mice. Doppler measurements of blood flow across the mitral valve showed a detrimental increase in the myocardial performance index (MPI, a measure of combined systolic and diastolic function) in 10-week-old male SMGRKO mice (*P* < 0.05), with a strong trend for an increase in MPI in females (*P* = 0.062, Fig. 2). Assessment of the component parts of the MPI showed that elongation of the isovolumetric contraction time underlies the elevation in MPI in both male and female SMGRKO mice (Fig. 2B and Supplementary Table 2). Thus, the increase in LV isovolumetric contraction time previously observed in SMGRKO mice *in utero* (Rog-Zielinska *et al*. 2013) persists into adulthood. In contrast, there were no genotype differences in E/A wave ratio (the ratio of the early (E) to the later atrial (A) ventricular filling velocities), early diastolic deceleration or mitral deceleration index (MDI) in adulthood (Supplementary Table 2), suggesting that the impairment in heart function is restricted to the early phase of contraction. Stroke volume was higher in male SMGRKO mice compared with that in controls (Supplementary Table 3), but this likely reflects a difference in heart size (see below), rather than function, as ejection fraction was equivalent between SMGRKO mice and controls, for both males and females (Supplementary Table 3).

Glucocorticoids can influence vascular tone (Whitworth *et al*. 1989, Pirpiris *et al*. 1992, Goodwin *et al*. 2008) and both hypothalamic–pituitary–adrenal axis activity and blood pressure are elevated in mice that are GR haploinsufficient (Michailidou *et al*. 2008). In SMGRKO mice, mean systolic blood pressure did not differ from controls (Supplementary Tables 4 and 5), consistent with previous findings in mice with VSM deletion of GR (Goodwin *et al*. 2008). Similarly, there were no genotype differences in plasma corticosterone levels (morning and evening) or adrenal gland weight (Supplementary Tables 4 and 5).

### Cardiac remodelling in SMGRKO mice

**Figure 3:**
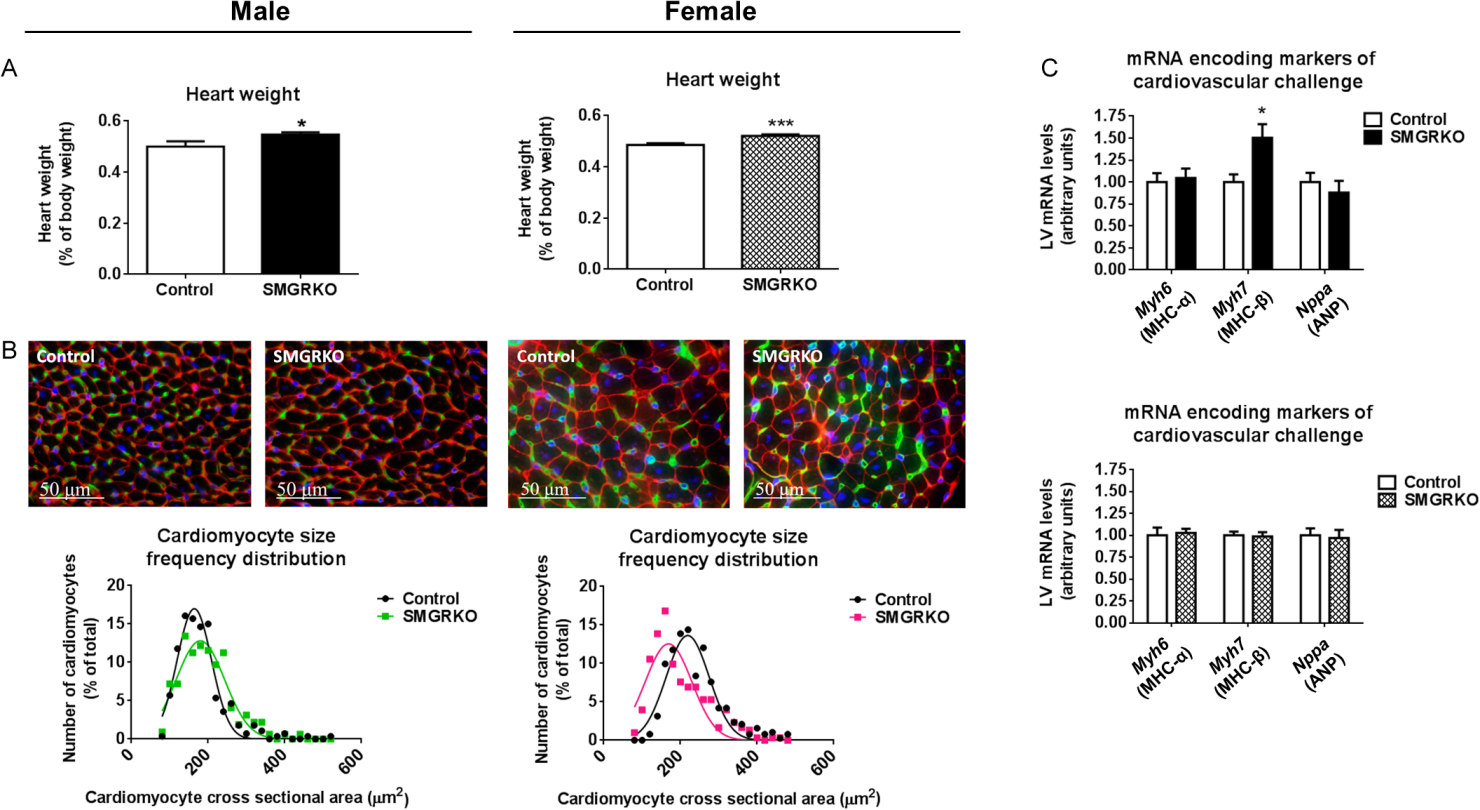
Heart weight is elevated in SMGRKO mice, with male SMGRKO mice showing evidence of pathological cardiomyocyte hypertrophy. Hearts of 12-week-old SMGRKO mice (black/hatched bars) and control littermates (white bars) were examined. (A) Heart weight (normalised for body weight) is elevated in male and female SMGRKO mice, compared with their respective littermate controls (*N* = 9–11). (B) Representative images of formalin-fixed, paraffin-embedded sections of LV of male and female SMGRKO mice and littermate controls showing wheat germ agglutinin staining of plasma membrane (red), isolectin B4 staining of the vasculature (green) and DAPI staining of nuclei (blue). The frequency distribution for cardiomyocyte cross-sectional area is right shifted, towards larger cardiomyocytes, in male SMGRKO mice (green line) but left shifted, towards smaller cardiomyocytes, in female SMGRKO mice (pink line) vs their respective controls (black lines). *N* = 8–10; 40 cardiomyocytes measured/heart. (C) Levels of mRNA encoding markers indicative of pathological cardiac remodelling, including myosin heavy chain alpha (*Myh6*), myosin heavy chain beta (*Myh7*) and atrial natriuretic peptide (*Nppa*), were not altered in LV of female SMGRKO mice. In contrast, levels of mRNA encoding *Myh7* were elevated in LV of male SMGRKO mice. *N* = 8–12. Data are means ± s.e.m. and were analysed using an unpaired *t*-test, *P < 0.05, ***P < 0.001.

**Figure 4:**
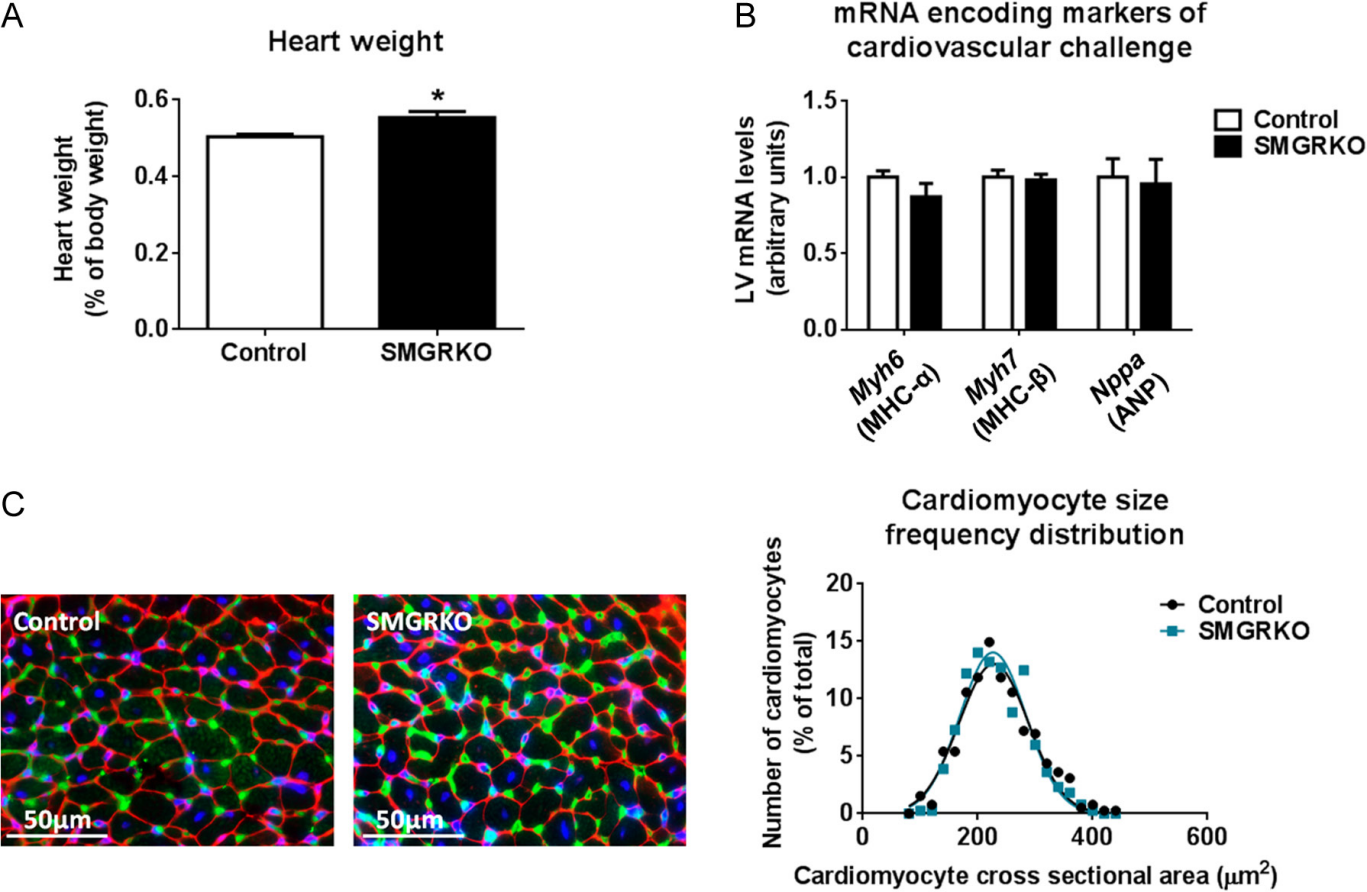
Heart weight is elevated in 6-week-old male SMGRKO mice, but there is no evidence of pathological cardiomyocyte hypertrophy. (A) Heart weight is elevated in 6-week-old male SMGRKO mice (black bar) compared with littermate controls (white bar) (*N* = 10–11). (B) Levels of mRNA encoding markers indicative of pathological cardiac remodelling, including myosin heavy chain alpha (*Myh6*), myosin heavy chain beta (*Myh7*) and atrial natriuretic peptide (*Nppa*), were not altered in LV of 6-week-old male SMGRKO mice compared with controls (*N* = 10–11). (C) Representative images of formalin-fixed, paraffin-embedded sections of LV of 6-week-old male SMGRKO mice and controls showing wheat germ agglutinin staining of plasma membrane (red), isolectin B4 staining of the vasculature (green) and DAPI staining of nuclei (blue). The frequency distribution for cardiomyocyte cross-sectional area did not differ in SMGRKO mice (blue line) compared with controls (black line). *N* = 10; 40 cardiomyocytes measured/heart. Data are means ± s.e.m. and were analysed using an unpaired *t*-test, *P < 0.05.

Heart weight was increased in male and female SMGRKO mice at 12 weeks of age (Fig. 3A). Body weight did not differ from controls (Supplementary Tables 4 and 5). To examine whether this associates with cellular hypertrophy, cardiomyocyte cross-sectional area was measured. Male SMGRKO mice showed a right shift in the cardiomyocyte cross-sectional area frequency distribution, towards larger cardiomyocytes, at 12 weeks (Fig. 3B and Supplementary Fig. 1A). Consistent with cardiomyocyte hypertrophy, levels of mRNA encoding myosin heavy chain (MHC) beta (*Myh7*), the foetal myosin heavy chain isoform and a marker of pathological hypertrophy (Swynghedauw 1986), were elevated in the LV of male mice at 12 weeks (Fig. 3C). In contrast, 12-week-old female SMGRKO mice showed a left shift in the cardiomyocyte cross-sectional area frequency distribution, towards smaller cardiomyocytes, (Fig. 3B and Supplementary Fig. 1A) and LV levels of mRNA encoding *Myh7* did not differ from controls (Fig. 3C). Moreover, cardiomyocyte volume, measured in female mice using the perforated patch technique to carry out voltage clamp on isolated cells, was similar between SMGRKO mice and their controls (Supplementary Fig. 1B). This confirms that the increase in heart weight in adult female mice is not due to cardiomyocyte hypertrophy. We next tested whether cardiomyocyte hypertrophy is present in younger male SMGRKO mice. Cardiomyocyte cross-sectional area in 6-week-old male SMGRKO mice was identical to that in control mice, and *Myh7* mRNA levels were normal, despite increased heart weight (Fig. 4), consistent with the idea that cardiomyocyte hypertrophy is not the primary cause of the elevated heart weight in male or female SMGRKO mice. Increased cardiomyocyte cross-sectional area associated with elevated levels of *Myh7* in 12-week-old male SMGRKO mice suggests cardiomyocyte hypertrophy arises secondarily, perhaps in response to the increase in cardiac workload at puberty, which is greater in males.

### SMGRKO mice develop LV cardiac fibrosis

**Figure 5:**
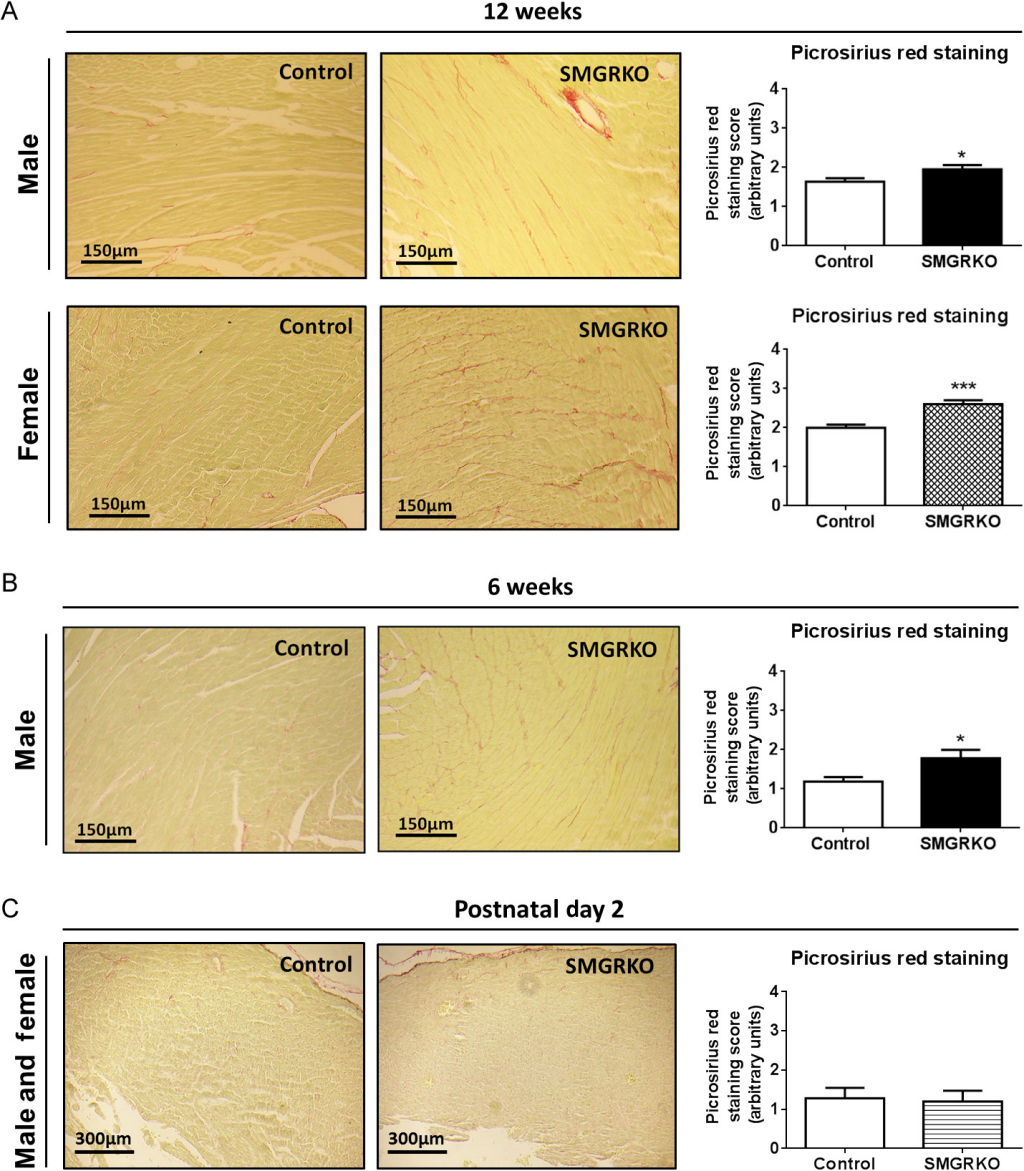
SMGRKO mice develop left ventricular cardiac fibrosis. Representative images of picrosirius red/fast green-stained sections of formalin-fixed paraffin-embedded hearts from SMGRKO mice and littermate controls (A, B and C). Assessment of picrosirius red staining of collagen in LV of SMGRKO mice (black/hatched/striped bars) compared with controls (white bars). Four fields of view were scored from the LV of each mouse by an investigator blind to genotype (scoring system described in ‘Materials and methods’ section). (A) 12-week-old males and females (*N* = 7–10). (B) 6-week-old males (*N* = 10). (C) 2-day-old males and females (*N* = 8–10). Data are means ± s.e.m. and were analysed using unpaired *t*-tests. *P < 0.05, ***P < 0.001.

**Figure 6:**
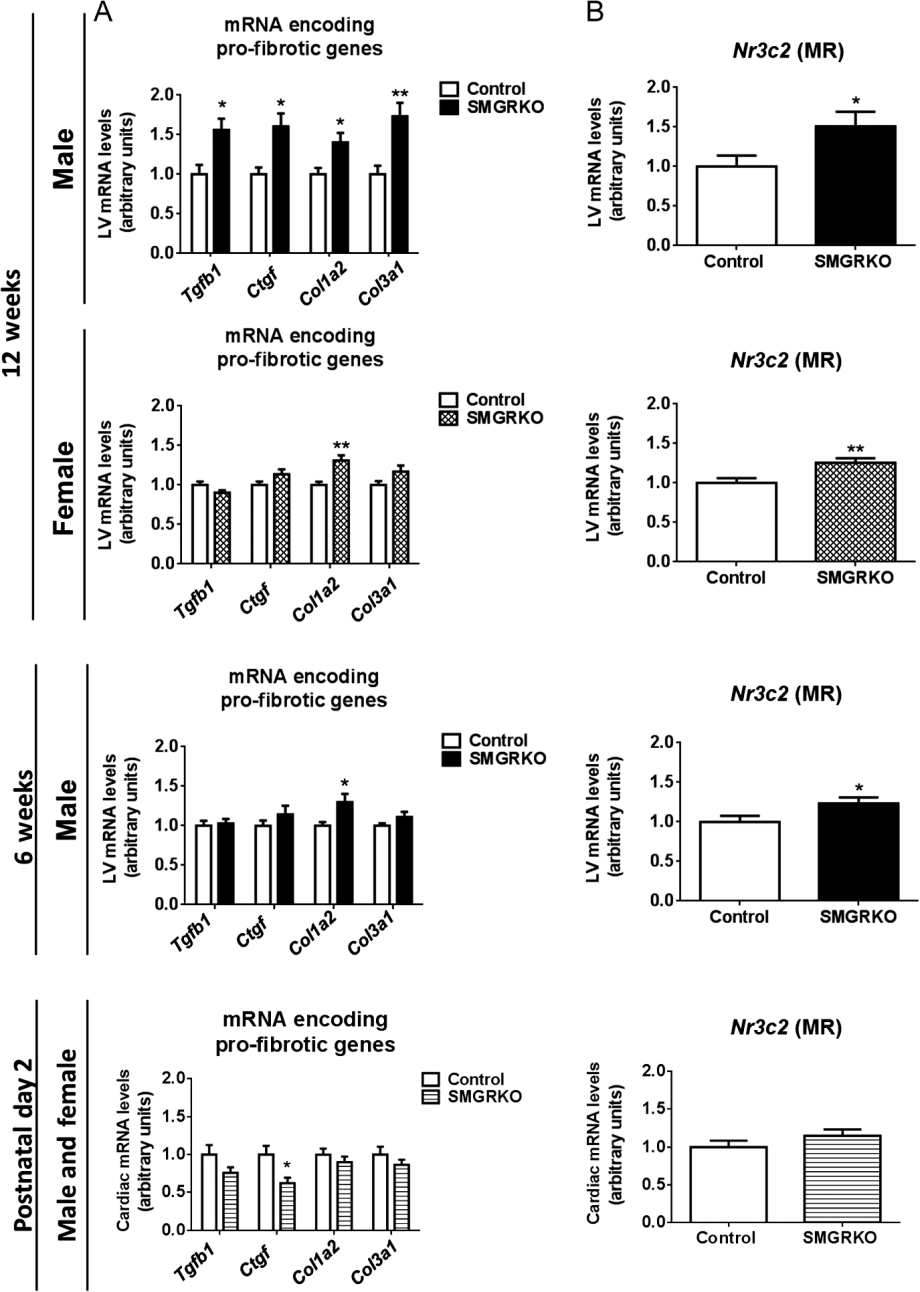
Levels of mRNA encoding pro-fibrotic genes are elevated in the LV of SMGRKO mice. (A) Quantitative RT-PCR measurements of mRNA show upregulation of pro-fibrotic genes, including transforming growth factor beta-1 (*Tgfb1*); connective tissue growth factor (*Ctgf*); collagen type 1α2 (*Col1a2*) and collagen type 3α1 (*Col3a1*) in LV of SMGRKO mice (black/hatched/striped bars) compared with controls (white bars). Measurements were made in male mice aged 6 weeks and both sexes at 12 weeks. Data are pooled from 2-day-old male and female mice. (B) Levels of *Nr3c2* mRNA (encoding MR) are elevated at 6 and 12 weeks, but not at 2 days, in LV of SMGRKO mice compared with controls. Data are means ± s.e.m. and were analysed using unpaired *t*-tests, *n* = 10–11, *P < 0.05, **P < 0.01.

To investigate whether the increased isovolumetric contraction time and/or cardiomyocyte hypertrophy in adult male SMGRKO mice are associated with cardiac fibrosis, picrosirius red was used to stain collagen. Compared to controls, staining of interstitial collagen was increased in hearts of 12-week-old male SMGRKO mice (Fig. 5A). Females also showed an increase in interstitial collagen (Fig. 5A). The increase in collagen staining in both genders suggests it is not related to cardiomyocyte hypertrophy, which only occurred in males. To test whether pro-fibrotic signalling is elevated in adult SMGRKO mice, RNA from LV of 12-week-old males and females was subject to profiling using a Qiagen Mouse Fibrosis RT² Profiler PCR Array. Levels of mRNA encoding pro-fibrotic markers and extracellular matrix remodelling proteins were increased in SMGRKO mice compared with controls (Supplementary Table 6). Levels of mRNA encoding platelet-derived growth factor alpha (*Pdgfa*), implicated in fibroblast proliferation, and alpha smooth muscle actin (*Acta2*), a marker of myofibroblast activation were also elevated (Supplementary Table 6). Quantitative RT-PCR confirmed higher levels of mRNA encoding transforming growth factor beta-1 (*Tgfb1*), connective tissue growth factor (*Ctgf*), collagen type 1α2 (*Col1a2*) and collagen type 3α1 (*Col3a1*) in LV of 12-week-old male SMGRKO mice compared to controls (Fig. 6A), though only the increase in *Col1a2* mRNA levels was significant in 12-week-old female SMGRKO mice. Similarly, quantitative RT-PCR confirmed higher expression of matrix remodelling genes in LV of adult male and female SMGRKO mice (Supplementary Fig. 2). In both genders, this was associated with elevated levels of *Nr3c2* mRNA, encoding MR. This suggests elevated MR signalling, possibly to compensate for reduced GR signalling, may drive the pro-fibrotic cardiac phenotype of SMGRKO mice. Expression of cytokines and chemokines associated with inflammation, including tumour necrosis factor (*Tnf*) and interleukin 1 beta (*Il1b*), remained unchanged in LV of SMGRKO mice (Supplementary Table 6). Thus, fibrosis does not appear to be secondary to ongoing inflammation. Picrosirius red staining showed interstitial collagen levels were already raised in LV of 6-week-old male SMGRKO mice (Fig. 5B), and this was again associated with elevated levels of *Col1a2* (Fig. 6A) and *Nr3c2* mRNA (Fig. 6B). Importantly, there was no increase in cardiac collagen staining, pro-fibrotic markers or *Nr3c2* mRNA levels in 2-day-old SMGRKO mice (Figs 5C and 6) suggesting that the fibrotic phenotype develops after the neonatal period.

### Spironolactone attenuates pro-fibrotic gene expression but does not prevent cardiac fibrosis in SMGRKO mice

**Figure 7:**
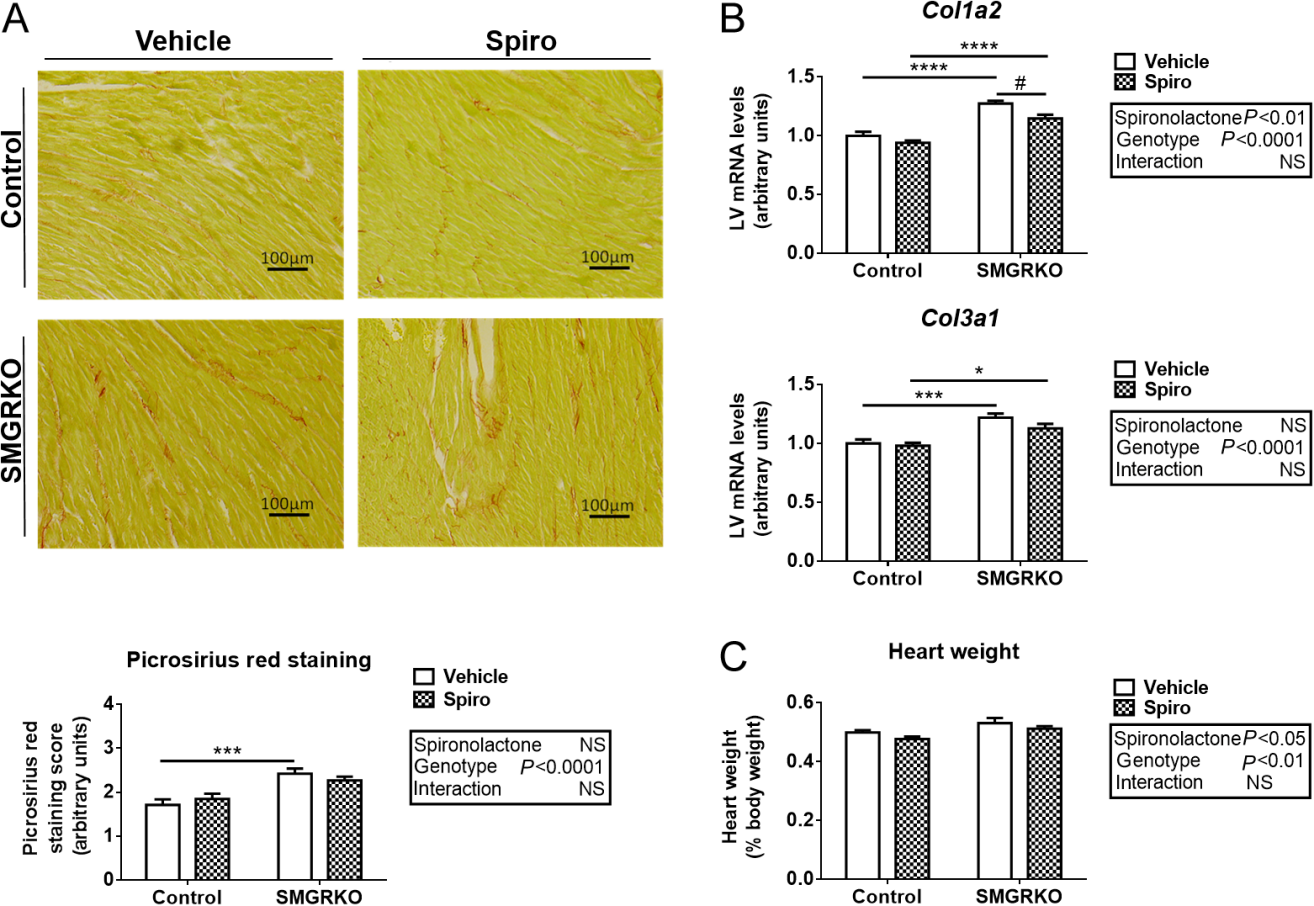
Spironolactone attenuates the increase in pro-fibrotic gene expression in male SMGRKO mice. Representative images of picrosirius red/fast green staining of sections of formalin fixed paraffin embedded hearts from male SMGRKO mice and control littermates treated from birth with the MR antagonist spironolactone or vehicle. Assessment of picrosirius red staining in 8-week-old mice shows that spironolactone treatment (chequered bars) did not prevent the increase in interstitial collagen in LV of SMGRKO mice compared to vehicle-treated controls (white bars). 4 fields of view were scored from the LV of each mouse by an investigator blind to genotype (scoring system described in ‘Materials and methods’ section) (*N* = 10–11). (B) Levels of mRNA-encoding collagen type 1α2 (*Col1a2*) and collagen type 3α1 (*Col3a1*) were measured in LV of 8-week-old mice. Spironolactone attenuated the increase in *Col1a2* mRNA levels in SMGRKO mice (*N* = 10–13). (C) Heart weight, expressed relative to body weight, was increased in 8-week-old male SMGRKO mice compared with controls, irrespective of spironolactone treatment. Spironolactone reduced heart weight in both genotypes compared with vehicle treated mice (*N* = 10–18). Data are means ± s.e.m. and were analysed by two-way ANOVA (boxes) followed by Tukey’s multiple comparisons test, * or ^#^*P* < 0.05, **P < 0.01, ***P < 0.001, ****P < 0.0001. *Genotype effect relative to control. ^#^Spironolactone effect, relative to vehicle.

To test whether MR antagonism can alleviate the pathological cardiac phenotype of SMGRKO mice, spirono­lactone was administered from birth. Spironolactone treatment decreased renal expression of the classical corticosteroid target genes, Serine/threonine-protein kinase (*Sgk1*) (Ueda *et al*. 2014) *and Fkbp5* in both genotypes (Supplementary Fig. 3A and B), confirming MR antagonism. Spironolactone did not prevent the development of cardiac fibrosis in male SMGRKO mice (Fig. 7A and B) though it did modestly attenuate the increase in *Col1a2* mRNA levels, whilst having no effect in control mice (Fig. 7B). This suggests that cardiac fibrosis in SMGRKO mice can be only partly attributed to activation of MR. Interestingly, although spironolactone treatment had no effect on the age-related increase in body weight of either SMGRKO or control mice (as expected) (Supplementary Fig. 3C), it caused a modest reduction in heart weight at 8 weeks of age compared with vehicle treatment, irrespective of genotype (Fig. 7C and Supplementary Fig. 3D). This implicates a role for MR signalling in postnatal cardiac growth independent of GR.

## Discussion

Our findings suggest complex roles for GR signalling in cardiac function, growth and remodelling. Whether these are interdependent or not is currently unclear. The modest impairment in systolic function in adult male and female SMGRKO mice, reflected in the elongated isovolumetric contraction time, is already present in foetal life when SMGRKO heart size is normal (Rog-Zielinska *et al*. 2013). Whilst the mechanism underlying the increase in isovolumetric contraction time is currently unknown, these findings suggest impaired systolic function is a primary and early manifestation of GR deficiency in cardiomyocytes, whereas the increase in heart size arises later. Whether aspects of the phenotype, particularly cardiac fibrosis, arise secondarily to the impaired systolic function is an important question for the future. It is also possible that the elongated isovolumetric contraction time (and/or cardiac immaturity, observed in late gestation SMGRKO foetuses (Rog-Zielinska *et al*. 2013)) may be causally related to the reduction in perinatal survival of SMGRKO mice.

Surviving SMGRKO mice have enlarged hearts. The increase in heart weight in the absence of cardiomyocyte hypertrophy in female and in young male SMGRKO mice suggests an increase in cell number, either cardiomyocytes or fibroblasts may contribute to the increase in heart weight. This most likely arises postnatally as foetal SMGRKO hearts are the same size as those of control mice at E17.5 (Rog-Zielinska *et al*. 2013). A role for GR in controlling heart growth in infancy is inferred by the observation that a GR gene polymorphism, linked with relative glucocorticoid resistance, associates with increased LV mass and systolic blood pressure in childhood (Geelhoed *et al*. 2011). SMGRKO mice have normal blood pressure, suggesting their increased heart weight is not a result of hypertension. Plausibly, the cardiac hypertrophy of SMGRKO mice may reflect greater cellularity. This could potentially arise from a prolonged period of cardiomyocyte proliferation in neonates, thereby increasing cardiomyocyte endowment. Consistent with this notion, preliminary data in hearts of 2-day-old SMGRKO mice showed an increase in the number of Ki67^+^ cardiomyocytes, compared to littermate controls (Supplementary Fig. 4) suggesting increased or prolonged proliferation. Interestingly, spironolactone antagonism of MR reduced heart weight in control as well as SMGRKO mice, suggesting MR activation normally promotes heart growth. However, as knock-out of MR in cardiomyocytes has little effect on the basal heart phenotype (Fraccarollo *et al*. 2011, Lother *et al*. 2011), this effect may be mediated indirectly. Nevertheless, previous data support a role for MR in early life cardiac growth. In neonatal rats, spironolactone was found to impair cardiac growth by reducing the number of proliferating cardiomyocytes (Sohn *et al*. 2010). In contrast, activation of MR by cortisol in foetal sheep increased cardiac cell proliferation (Feng *et al*. 2013). Given the critical role of GR signalling in cardiac maturation (Rog-Zielinska *et al*. 2014*b*), it is possible that the GR:MR balance may determine the effect of corticosteroids on cardiac growth in early life. This may, in turn, have consequences for cardiac health in adulthood. Whether GR activation (or the GR:MR balance) limits early heart growth (and cardiomyocyte endowment) by promoting the neonatal switch in cardiomyocytes from proliferative to hypertrophic growth merits investigation in the future.

Cardiomyocyte hypertrophy, associated with elevated LV expression of *Myh7*, occurred in adult male but not female SMGRKO mice. Moreover, it only became manifest in male SMGRKO mice after puberty. It is well known that adult male hearts are hypertrophied relative to female, with this ascribed to direct effects in cardiomyocytes as a result of the rise in androgen levels at puberty in males (Koenig *et al*. 1982, de Simone *et al*. 1995, Marsh *et al*. 1998, Leinwand 2003). Myocardial remodelling in response to pathological or physiological stimuli also differs between male and female hearts (Piro *et al*. 2010). In rats, when cardiac remodelling was induced by pressure overload, there was a greater increase in mRNA encoding *Myh7* in the LV of males than that in females (Weinberg *et al*. 1999). Our data are consistent with this gender difference in susceptibility to cardiac remodelling and suggest that GR deficiency in cardiomyocytes and/or vascular smooth muscle cells potentiates the hypertrophic actions of androgens in pubertal males. Whether SMGRKO mice show a gender-specific difference in models of hypertrophic cardiac disease, for example with pressure-overload, will be important to establish and may provide insight into the role glucocorticoid action plays in gender differences in susceptibility to heart disease.

Both male and female SMGRKO mice develop interstitial cardiac fibrosis. This arises postnatally, with no increase in pro-fibrotic gene signalling evident in neonates. In both genders, interstitial fibrosis is associated with increased expression of genes encoding collagen and matrix remodelling enzymes, as well as *Acta2*, a marker of myofibroblast activation (Darby *et al*. 1990). Interestingly, the array data showed gender differences in the level of expression of a number of genes associated with fibrosis, including *Acta2*, suggesting there may be a gender difference in fibrotic response to cardiac injury in these mice. MR expression was increased in hearts of adult SMGRKO mice. This is unlikely to be direct compensation for lack of GR, levels in neonatal hearts were normal and MR expression is reduced (rather than increased) in another GR-knockout model (Meijer *et al*. 1997). Elevated MR expression has been suggested to drive cardiac fibrosis (Lother *et al*. 2011), and MR antagonism is protective in murine models of pressure-overload (Kuster *et al*. 2005) and post-MI remodelling (Fraccarollo *et al*. 2003). However, antagonism of MR from birth had only a modest effect on cardiac fibrosis in SMGRKO mice, so whilst a possible contributor, MR activation does not appear to be the primary driver of the fibrosis. At present, it is unclear what the underlying cause is. There is no evidence of inflammation in the hearts of SMGRKO mice, which showed similar expression to control mice of a range of cytokine and chemokine mRNAs. The lack of overt inflammation is also consistent with unaltered circulating glucocorticoid levels in SMGRKO mice, which would be expected to increase with cardiac inflammation. Plausibly, the fibrosis could result from mechanical stress related to the elongated isovolumetric contraction time. Although little myocardial deformation occurs during isovolumetric contraction, there is circumferential stretch in the subepicardial layer and circumferential shortening in the midwall and subendocardial layer (Ashikaga *et al*. 2009). Foetal SMGRKO mice show altered cardiac ultrastructure, including the organisation of the outermost circumferential muscle layer. This suggests contractile abnormalities in cardiomyocytes *in utero* that persist into adulthood may activate repair pathways postnatally to cause fibrosis.

The cardiac phenotype of SMGRKO mice shows similarities, but also some differences, to that of mice with *MHCα-Cre* mediated deletion of GR in cardiomyocytes, termed CardioGRKO mice (Oakley *et al*. 2013). Similarities confirm the role of cardiomyocyte GR, whereas differences are likely to relate to temporal differences in *Cre* transgene expression or to the tissue specificity of the GR deletion: in cardiomyocytes and vascular smooth muscle in SMGRKO mice, but restricted to cardiomyocytes in CardioGRKO mice. Both CardioGRKO and SMGRKO mice show a relatively high mortality rate, but the timing differs. In both, females show a higher mortality than males. The normal Mendelian ratio of SMGRKO mice at E17.5 (Rog-Zielinska *et al*. 2013), but deviance from this following birth, suggests SMGRKO mice die in late gestation or around the time of birth (there are no excess deaths after the neonatal period). In contrast, CardioGRKO mice are born at the expected Mendelian ratio yet die prematurely of congestive heart failure. This difference in timing of death is likely to reflect the late gestation expression of the *MHCα-Cre* transgene in ventricular cardiomyocytes (Lyons *et al*. 1990), compared to the earlier and transient expression of the *Sm22α-Cre* transgene. Thus, CardioGRKO mice may retain sufficient GR in cardiomyocytes to survive the high demand on the heart during and shortly after birth, whereas SMGRKO mice appear vulnerable at birth.

Both SMGRKO and CardioGRKO mice have larger hearts than their respective controls. Cardiomyocyte hypertrophy occurred in adult SMGRKO and CardioGRKO mice, associated with an increase in *Myh7* mRNA. In SMGRKO mice, this was restricted to males (the gender of CardioGRKO mice was not specified). In CardioGRKO mice, cardiomyocyte hypertrophy preceded heart failure and premature death (Oakley *et al*. 2013), but there was no excess mortality in SMGRKO mice after the immediate neonatal period. The lack of progression to heart failure in SMGRKO mice suggests that heart failure is not an inevitable consequence of GR deficiency in cardiomyocytes. It remains possible that the SMGRKO mice that survive to weaning and the CardioGRKO mice that do not go on to develop heart failure have residual GR expression in cardiomyocytes. Alternatively, GR deficiency may potentiate the genotoxic effects associated with the continuing high expression of *MHCα-Cre* (Buerger *et al*. 2006) in CardioGRKO mice, which are otherwise only apparent at a sub-clinical level. In contrast, *Sm22α-Cre* is only transiently expressed in cardiomyocytes during development and Cre is absent in adult cardiomyocytes (Li *et al*. 1996). Whilst we have not formally ruled out a postnatal cardiac phenotype solely due to transient expression of Cre during development, we have used the same line of *SM22-Cre* transgenic mice to disrupt *Hsd11b1* expression, with no discernible effect on either cardiac size or the response to cardiac injury (White *et al*. 2015).

CardioGRKO mice showed a strong cardiac inflammatory response by 2 months of age, which we did not observe in SMGRKO mice. In contrast, SMGRKO mice showed a fibrotic response, not observed in CardioGRKO mice. These differences are likely to relate to the *Cre* used to delete GR in cardiomyocytes. It is possible that GR deletion in other cell types such as vascular smooth muscle cells is responsible for the cardiac fibrosis of SMGRKO mice. Interestingly, expression of *Acta2*, a marker of myofibroblast activation, was increased in CardioGRKO mice as well as SMGRKO mice, though in CardioGRKO mice this was not associated with elevated collagen expression. In contrast, the lack of evidence for an inflammatory response in hearts of SMGRKO mice suggests that the inflammation seen in CardioGRKO mice is secondary to the pathological cardiomyocyte changes that lead to heart failure in these mice.

Recent studies have made progress in elucidating the individual roles of GR and MR signalling in specific cardiovascular cell types (Oakley & Cidlowski 2015, Young & Rickard 2015, Richardson *et al*. 2016). Here, we have provided further evidence for a critical requirement for GR in myocytes in the cardiovascular system. SMGRKO mice may prove to be a useful new model to investigate the mechanisms that give rise to cardiac fibrosis without alteration in blood pressure or invoking pharmacological activation of MR. The absence of maladaptive cardiomyocyte hypertrophy in female SMGRKO mice highlights the necessity to investigate sex differences in cardiovascular research studies. Future research needs to address the relationship between MR and GR signalling to elucidate the consequences of alterations in the GR:MR balance across the range of cardiac cell types. This will provide essential insight into the cell-specific role of corticosteroids, both in early life programming of cardiomyocyte endowment and in subsequent cardiac pathology, in order to open up avenues for therapeutic manipulation of corticosteroid signalling.

## Supplementary Material

Supporting Figure 1

Supporting Figure 2

Supporting Figure 3

Supporting Figure 4

Table S1 **Primer sequences and probes used for RT-PCR.** Primers were designed using the Roche Applied Science Universal Probe Library Assay Design Centre and tested for specificity.

Table S2 Echocardiographic Doppler measurements of blood flow across the mitral valve of 10 week old male SMGRKO mice and littermate controls. Values are means ± SEM with number indicated in brackets. *p<0.05, **p<0.01 (Unpaired t-test).

Table S3 Echocardiography measurements obtained from the parasternal long axis in SMGRKO mice and control littermates aged 10 weeks. Echocardiography parameters were obtained from measurements made from the parasternal long-axis view in B-Mode and M-Mode. Data are means ± SEM and were analysed using an unpaired t-test, n=10-17 (indicated in brackets), *p<0.05.

Table S4 **Physiological parameters for male SMGRKO mice and littermate controls at 12 weeks of age.** Values are means ± SEM with number indicated in brackets. Data were analysed by unpaired t-test.

Table S5 Physiological parameters for female SMGRKO mice and littermate controls at 12 weeks of age. Values are means ± SEM with number indicated in brackets. ****p<0.0001. Data were analysed by unpaired t-test.

Table S6 Mouse Fibrosis PCR Array data. Mean values are 2-^ΔCt^ where ΔC_t_ = C_t_ gene of interest - C_t_ house-keeper gene. The average house-keeper C_t_ was taken from a panel of genes (*Gusb, Actb, Gapdh, Hprt and Hsp90ab1*). N=8 per group. Data were analysed by two-way ANOVA, *p<0.05, **p<0.01, ***p<0.001, ****p<0.0001

## Declaration of interest

The authors declare that there is no conflict of interest that could be perceived as prejudicing the impartiality of the research reported.

## Funding

This work was supported by the Medical Research Council (studentship awarded to R V R, project grant MR/P002811/1), a studentship from the British Heart Foundation (E J B) and a Society for Endocrinology Early Career grant (R V R). Additional support was provided by the Centre for Cardiovascular Science, University of Edinburgh.

## Author contribution statement

Conception: R V R, K E C and E R-Z. Design: R V R, C J K, G A G and K E C. Execution: R V R, E J B, R D, X P, E R-Z, W W, K S, A J W T, E A S A, M E D and C J K. Interpretation: R V R, M E D, C M M, C J K, G A G and K E C. Manuscript preparation: R V R, K E C, E J B, G A G and E R-Z.
