# Supplementary figures and images for: Glucocorticoid receptor alters isovolumetric contraction and restrains cardiac fibrosis
Source: J Endocrinol. 2017 Jan 5;232(3):437–50. doi: 10.1530/JOE-16-0458 (PMC5292999; doi:10.1530/JOE-16-0458)

**A.**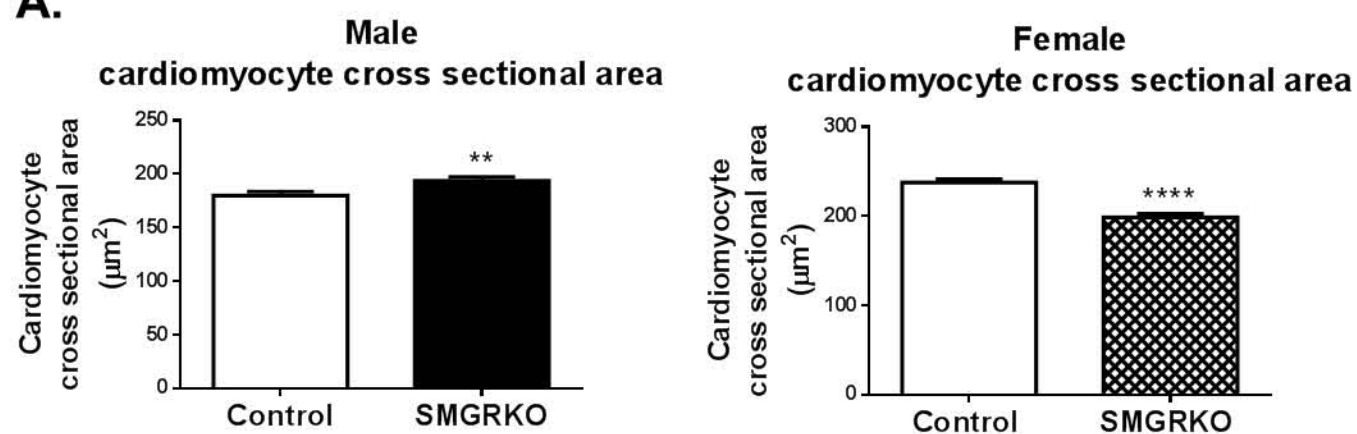**B.**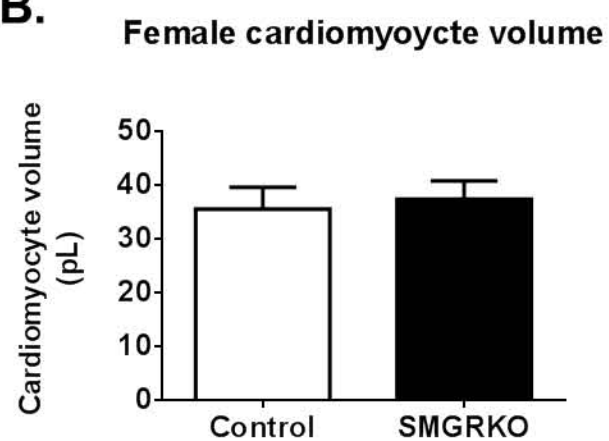

Supplement: Supporting Figure 1 [file joe-232-351-s001.pdf]

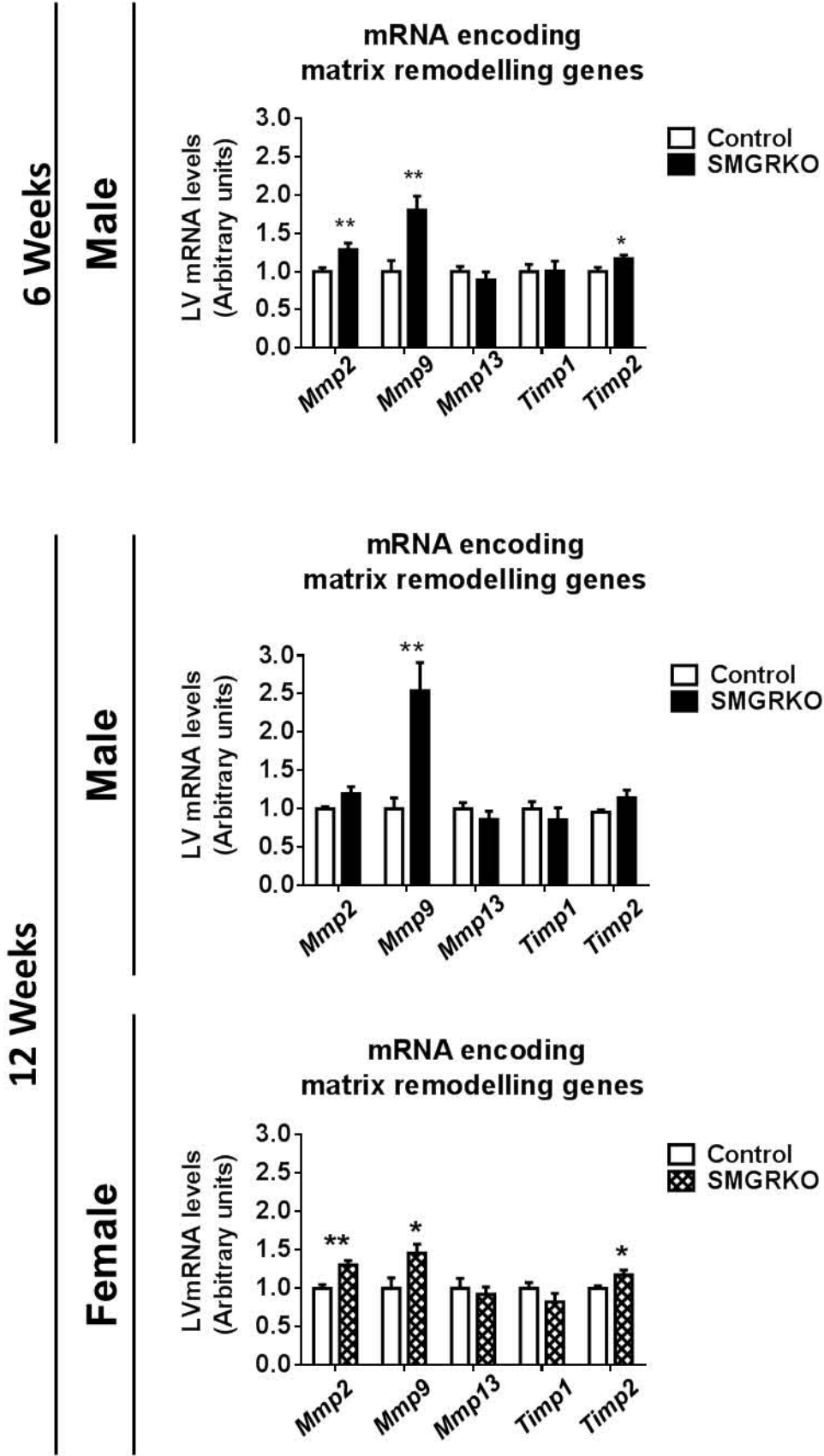

Supplement: Supporting Figure 2 [file joe-232-351-s002.pdf]

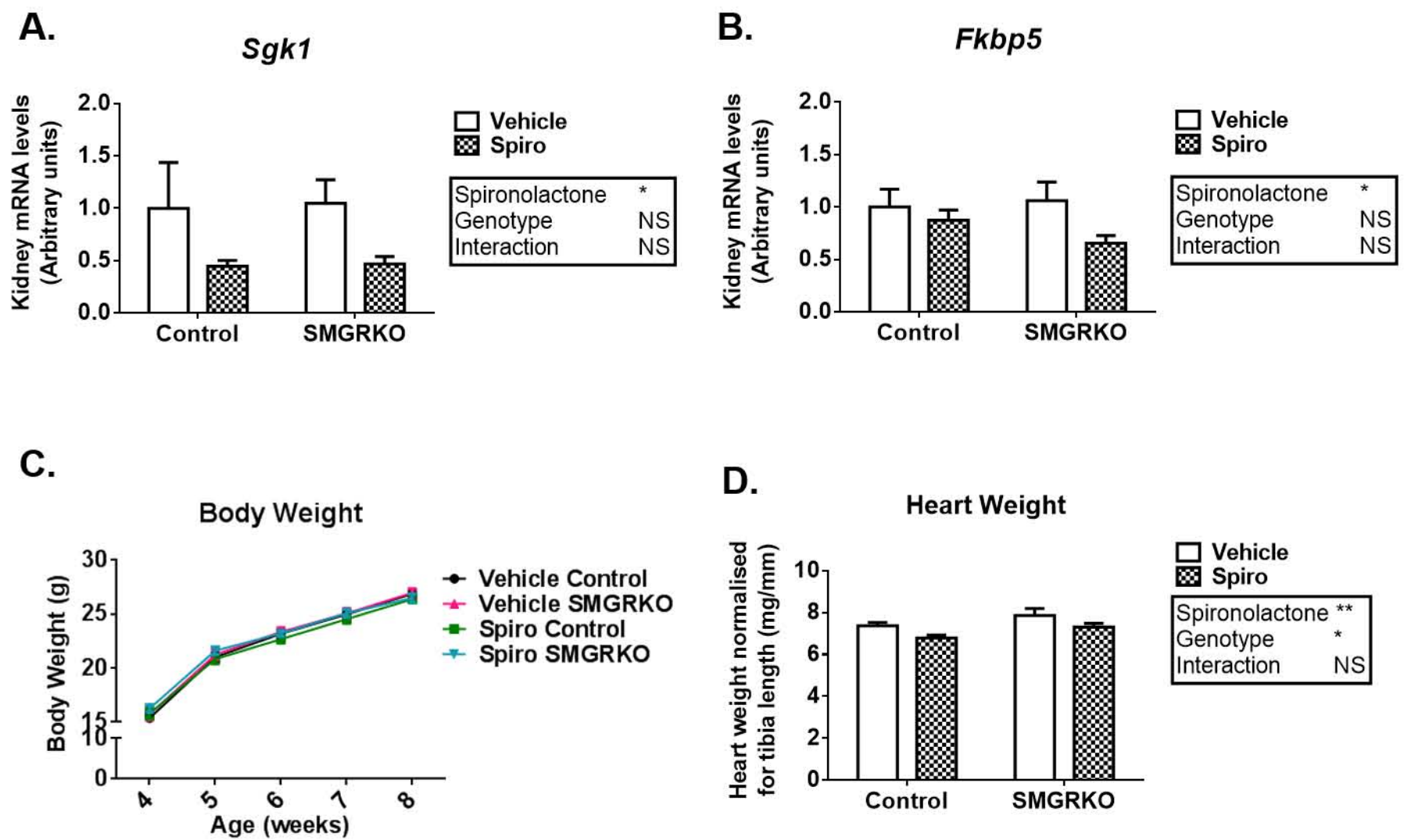

Supplement: Supporting Figure 3 [file joe-232-351-s003.pdf]

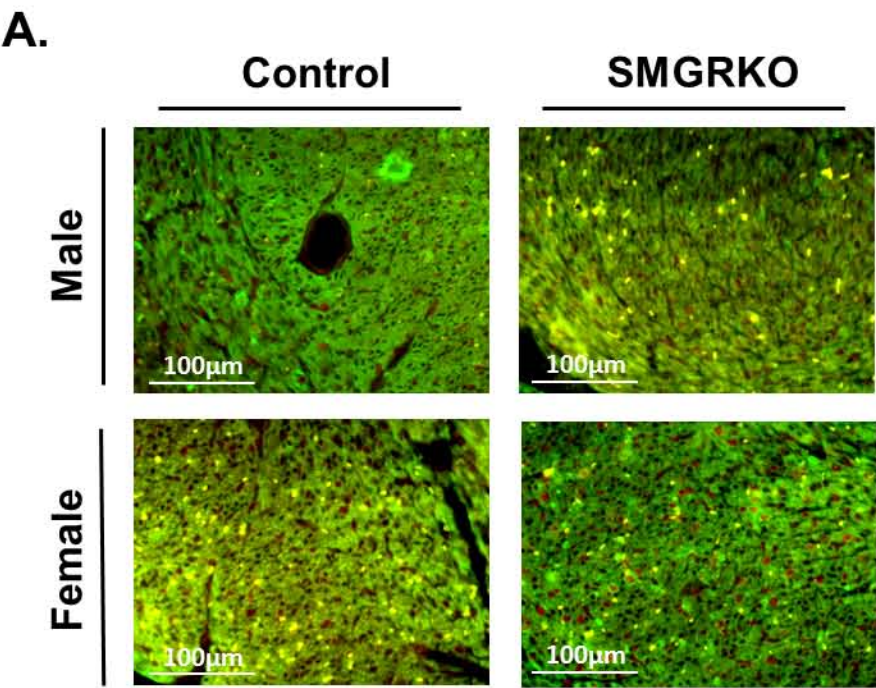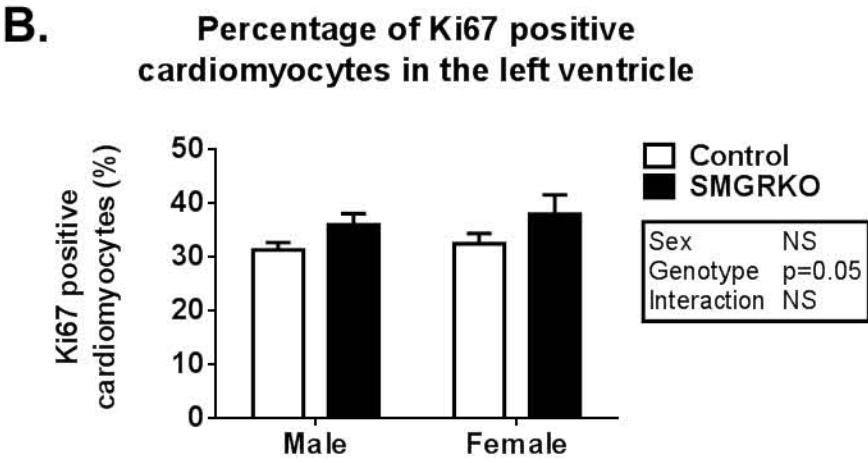

Supplement: Supporting Figure 4 [file joe-232-351-s004.pdf]
