# Supplementary material for: Glucocorticoid receptor alters isovolumetric contraction and restrains cardiac fibrosis
Source: J Endocrinol. 2017 Jan 5;232(3):437–50. doi: 10.1530/JOE-16-0458 (PMC5292999; doi:10.1530/JOE-16-0458)
Supplement: Table S1 [file joe-232-351-t001.pdf]

Supplementary Table 1. Primer sequences and probes used for RT-PCR.
Primers were designed using the Roche Applied Science Universal Probe Library Assay Design Centre and tested for specificity.

Primer Name	Sequence 5'-3'		Probe #
	Forward	Reverse	
Actb	ctaaggccaaccgtgaaaag	accagaggcatacagggaca	64
Nppa	cacagatctgatggatttcaaga	cctcatcttctaccggcatc	25
Ctgf	tgacctggaggaaaacattaaga	agccctgtatgtcttcacactg	71
Col1a2	gcaggttcacctactctgtcct	cttgccccattcatttgtct	46
Col3a1	tcccctggaatctgtgaatc	tgagtcgaattggggagaat	49
Fkbp5	aaacgaaggagcaacggtaa	tcaaatgtccttccaccaca	97
Gapdh	gggttcctataaatacgcactgc	ccattttgtctacgggacga	52
Nr3c1	caaagattgcaggtatcctatgaa	cttggctcttcagaccttcc	91
Nr3c2	ccctaccatgtcctagaaaagc	agaacgctccaaggtctgag	109
Myh6	cgcatcaaggagctcacc	cctgcagccgcattaagt	6
Myh7	gcatctggaaattccgttagg	ggctcgtcatccttattagacc	16
Mmp2	taacctggatgccgtcgt	tcaggtaataagcacccttgaa	77
Mmp9	acgacatagacggcatcca	gctgtggttcagttgtggtg	19
Mmp13	gccagaacttcccaaccat	tcagagcccagaattttctcc	89
Sgk1	gattgccagcaacacctatg	ttgatttgttgagagggacttg	91
Tgfb1	tggagcaacatgtggaactc	cagcagccggttaccaag	72
Timp1	tcagagcccagaattttctcc	agggatagataaacagggaaacact	76
Timp2	cgttttgcaatgcagacgta	ggaatccacctccttctcg	21

#
