# Supplementary material for: Glucocorticoid receptor alters isovolumetric contraction and restrains cardiac fibrosis
Source: J Endocrinol. 2017 Jan 5;232(3):437–50. doi: 10.1530/JOE-16-0458 (PMC5292999; doi:10.1530/JOE-16-0458)
Supplement: Table S2 [file joe-232-351-t002.pdf]

Supplementary Table 2. Echocardiographic Doppler measurements of blood flow across the mitral valve of 10 week old male SMGRKO mice and littermate controls. Values are means ± SEM with number indicated in brackets. *p<0.05, **p<0.01 (Unpaired t-test).

Doppler measurement (Unit)	Sex	Control	SMGRKO	
Peak E Wave Velocity (mm/s)	Male	860±37 (11)	918±32 (16)	
	Female	955±23 (11)	891±38 (11)	
Peak A Wave Velocity(mm/s)	Male	580±24 (11)	638±24 (15)	
	Female	485±27 (12)	416±28 (11)	
E/A Wave Ratio	Male	1.50±0.07 (11)	1.42±0.06 (14)	
	Female	1.92±0.09 (11)	2.10±0.11 (10)	
E wave Deceleration (mm/s^2)	Male	32567±1925 (10)	36654±1618 (16)	
	Female	39712±1749 (12)	44320±3865 (11)	
MDI = E wave Decel. Time (ms/[mm/s])	Male	0.031±0.002 (10)	0.029±0.001 (15)	
Peak E wave velocity	Female	0.025±0.001 (12)	0.022±0.002 (10)	
Pressure Half Time	Male	7.08±0.34 (10)	6.48±0.22 (14)	
	Female	6.26±0.27 (11)	5.56±0.33 (11)	
Myocardial Performance Index	Male	0.64+0.03 (11)	0.73±0.02 (16)	*
(Arbitrary units)	Female	0.56±0.03 (12)	0.65±0.03 (11)	
Isovolumetric Contraction Time (ms)	Male	12.9±0.8 (11)	15.5±0.6 (15)	*
	Female	11.8±0.5 (11)	14.8±0.8 (11)	**
Ejection Time (ms)	Male	42.7±1.1 (11)	40.44±0.82 (15)	
	Female	45.5±0.8 (11)	47.0±0.90 (10)	
Isovolumetric Relaxation Time (ms)	Male	14.4±1.1 (11)	13.7±0.4 (16)	
	Female	13.5±0.5 (12)	15.2±0.7 (10)	
Heart Rate (BPM)	Male	482±17 (11)	464±14 (15)	
	Female	454±9 (12)	420±8 (10)	*

#
