# Supplementary material for: Glucocorticoid receptor alters isovolumetric contraction and restrains cardiac fibrosis
Source: J Endocrinol. 2017 Jan 5;232(3):437–50. doi: 10.1530/JOE-16-0458 (PMC5292999; doi:10.1530/JOE-16-0458)
Supplement: Table S3 [file joe-232-351-t003.pdf]

Supplementary Table 3. Echocardiography measurements obtained from the parasternal long axis in SMGRKO mice and control littermates aged 10 weeks. Echocardiography parameters were obtained from measurements made from the parasternal long-axis view in B-Mode and M-Mode. Data are means ± SEM and were analysed using an unpaired t-test, n=10-17 (indicated in brackets), *p<0.05.

**Echocardiography Parameter (Units; Acquisition Mode)**	**Sex**	**Control**	**SMGRKO**	
LV End-Systolic Area (mm^2;B)	Male	15.8±1.2 (12)	15.3±0.7 (17)	
	Female	11.3 ±0.8 (12)	10.1± 0.7 (10)	
LV End-Diastolic Area (mm^2;B)	Male	24.0±1.0 (12)	24.9±0.7 (17)	
	Female	20.4±0.8 (12)	19.3± 10.5 (10)	
Endocardial Area Change (mm^2;B)	Male	8.38±0.41 (12)	9.63±0.39 (17)	*
	Female	8.76± 0.56 (12)	8.95± 0.42 (10)	
Endocardial %Fractional Area Change (%;B)	Male	35.5±2.3 (12)	37.7±.1.3 (16)	
	Female	43.7±2.5 (12)	46.6± 2.3 (10)	
Ejection Fraction (%;B)	Male	57.2±2.9 (11)	57.4±1.7 (16)	
	Female	64.8± 2.8 (12)	68.4± 2.9 (10)	
Stroke Volume (µl;B)	Male	32.3±1.7 (12)	38.2±1.8 (16)	*
	Female	30.5± 1.8 (12)	29.0± 1.3 (10)	
Cardiac Output (ml/min;B)	Male	15.5±0.7 (11)	18.7±0.9 (17)	*
	Female	13.7± 0.9 (12)	12.8± 0.6 (9)	
LV Wall Thickness (mm;B)	Male	0.95±0.04 (12)	0.86±0.02 (17)	*
	Female	0.80±0.02 (12)	0.83± 0.02 (10)	
Left Ventricle Anterior Wall; systole (mm;M)	Male	1.24±0.11 (11)	1.15±0.05 (16)	
	Female	1.51±0.07 (12)	1.72±0.07 (10)	
Left Ventricle Anterior Wall; diastole (mm;M)	Male	0.82±0.06 (11)	0.79±0.03 (16)	
	Female	0.960.02 (12)	1.03±0.05 (10)	
Left Ventricle Internal Diameter; systole (mm;M)	Male	2.23±0.13 (11)	2.45±0.11 (16)	
	Female	1.95± 0.11 (12)	1.67± 0.11(10)	
Left Ventricle Internal Diameter; diastole (mm;M)	Male	3.70±0.08 (11)	3.91±0.05 (15)	*
	Female	3.41± 0.08 (12)	3.27± 0.07(10)	
Left Ventricle Paraventricular Wall; systole (mm;M)	Male	1.43±0.05 (10)	1.44±0.07 (16)	
	Female	1.21± 0.12 (12)	1.45± 0.06 (10)	
Left Ventricle Paraventricular Wall ; diastole (mm;M)	Male	0.90±0.06 (10)	0.90±0.05 (15)	
	Female	0.83± 0.08 (12)	1.03± 0.07 (10)	
Fractional Shortening (%;M)	Male	40.0±2.9 (12)	37.5±2.0 (16)	
	Female	43.2± 2.2 (12)	49.3± 2.7 (10)	
Left Ventricle Mass (mg;M)	Male	100.4±10.9 (11)	96.0±4.2 (15)	
	Female	86.5± 6.9 (12)	98.5± 6.0 (10)	

#
