# Supplementary material for: Glucocorticoid receptor alters isovolumetric contraction and restrains cardiac fibrosis
Source: J Endocrinol. 2017 Jan 5;232(3):437–50. doi: 10.1530/JOE-16-0458 (PMC5292999; doi:10.1530/JOE-16-0458)
Supplement: Table S4 [file joe-232-351-t004.pdf]

Supplementary Table 4. Physiological parameters for male SMGRKO mice and littermate controls at 12 weeks of age. Values are means ± SEM with number indicated in brackets. Data were analysed by unpaired t-test.

**Parameter**	**Male Control**	**Male SMGRKO**
Mean systolic blood pressure (mmHg)	111±1 (9)	108±1 (6)
Body weight (g)	30.9±0.8 (9)	29.8±0.7 (12)
Kidney weight (% body weight)	0.57±0.02 (9)	0.54±0.02 (12)
Adrenal gland weight (% body weight)	0.0052±0.0002 (9)	0.0054±0.0002(12)
Heart weight (normalised for tibia length; mg/mm)	8.08±0.4 (9)	8.69±0.25 (11)
Plasma corticosterone am (nM)	156±28 (8)	147±18 (8)
Plasma corticosterone pm (nM)	879±82 (12)	749±169 (13)

#
