# Supplementary material for: Glucocorticoid receptor alters isovolumetric contraction and restrains cardiac fibrosis
Source: J Endocrinol. 2017 Jan 5;232(3):437–50. doi: 10.1530/JOE-16-0458 (PMC5292999; doi:10.1530/JOE-16-0458)
Supplement: Table S5 [file joe-232-351-t005.pdf]

Supplementary Table 5. Physiological parameters for female SMGRKO mice and littermate controls at 12 weeks of age. Values are means ± SEM with number indicated in brackets. ****p<0.0001. Data were analysed by unpaired t-test.

**Parameter**	**Female Control**	**Female SMGRKO**
Mean systolic blood pressure (mmHg)	115±2 (7)	119±2 (9)
Body weight (g)	21.8±0.6 (10)	23.1±0.3 (11)
Kidney weight (% body weight)	0.49±0.03 (10)	0.53±0.02 (11)
Adrenal gland weight (% body weight)	0.0103±0.0003 (10)	0.0112±0.0003 (10)
Heart weight (normalised for tibia length; mg/mm)	5.73±0.14 (10)	6.56±0.08 (10) ****

#
