# Supplementary material for: Glucocorticoid receptor alters isovolumetric contraction and restrains cardiac fibrosis
Source: J Endocrinol. 2017 Jan 5;232(3):437–50. doi: 10.1530/JOE-16-0458 (PMC5292999; doi:10.1530/JOE-16-0458)
Supplement: Table S6 [file joe-232-351-t006.pdf]

Supplementary Table 6. Mouse Fibrosis PCR Array data. Mean values are $2^{-\Delta Ct}$ where $\Delta C_t = C_t$ gene of interest - C_t house-keeper gene. The average house-keeper C_t was taken from a panel of genes (*Gusb, Actb, Gapdh, Hprt* and *Hsp90ab1*). N=8 per group. Data were analysed by two-way ANOVA, *p<0.05, **p<0.01, ***p<0.001, ****p<0.0001.

			Control			SMGRKO			2-way ANOVA		
			MEAN	SEM	N	MEAN	SEM	N	Interaction	Sex	Genotype
Actin, alpha 2, smooth muscle, aorta	Acta2	Male	0.322	0.04	8	0.528	0.034	8			
		Female	0.596	0.08	7	0.772	0.13	7	ns	**	*
Angiotensinogen (serpin peptidase inhibitor, clade A, member 8)	Agt	Male	0.00321	0.00022	8	0.00131	0.00016	8			
		Female	0.00417	0.0004	7	0.00137	0.000065	7	ns	*	****
Thymoma viral proto-oncogene 1	Akt1	Male	0.214	0.0065	8	0.218	0.017	8			
		Female	0.22	0.017	7	0.231	0.015	8	ns	ns	ns
B-cell leukemia/lymphoma 2	Bcl2	Male	0.0164	0.00086	8	0.0257	0.0014	8			
		Female	0.0197	0.0012	8	0.0245	0.0012	7	ns	ns	****
Bone morphogenetic protein 7	Bmp7	Male	0.00278	0.0003	8	0.00198	0.00025	8			
		Female	0.00262	0.00026	8	0.00194	0.00023	8	ns	ns	**
Caveolin 1, caveolae protein	Cav1	Male	0.38	0.025	8	0.383	0.033	8			
		Female	0.451	0.038	8	0.343	0.03	7	ns	ns	ns
Chemokine (C-C motif) ligand 11	Ccl11	Male	0.00498	0.00057	7	0.00323	0.00038	8			
		Female	0.00174	0.00033	8	0.00101	0.00021	8	ns	****	**
Chemokine (C-C motif) ligand 12	Ccl12	Male	0.00605	0.00054	7	0.0097	0.002	7			
		Female	0.0134	0.0025	8	0.00886	0.0015	7	*	ns	ns
Chemokine (C-C motif) ligand 3	Ccl3	Male	0.000524	0.000085	8	0.000589	0.0001	8			
		Female	0.000588	0.0001	7	0.000389	0.000079	7	ns	ns	ns
Chemokine (C-C motif) receptor 2	Ccr2	Male	0.00467	0.00048	8	0.00573	0.00081	8			
		Female	0.00396	0.00064	8	0.00296	0.00028	7	ns	**	ns
CCAAT/enhancer binding protein (C/EBP), beta	Cebpb	Male	0.06	0.0077	6	0.0463	0.0029	6			
		Female	0.0449	0.01	5	0.0488	0.0065	5	ns	ns	ns
Collagen, type I, alpha 2	Col1	Male	0.216	0.0079	7	0.244	0.015	7			
		Female	0.235	0.016	7	0.285	0.025	7	ns	ns	*
Collagen, type III, alpha 1	col3a1	Male	0.58	0.046	8	0.689	0.048	8			
		Female	0.706	0.049	7	0.891	0.064	7	ns	**	**
Connective tissue growth factor	CTGF	Male	0.183	0.011	7	0.223	0.044	8			
		Female	0.168	0.015	8	0.17	0.01	7	ns	ns	ns
Chemokine (C-X-C motif) receptor 4	Cxcr4	Male	0.0262	0.00076	7	0.0304	0.0036	8			
		Female	0.0301	0.0029	8	0.0274	0.0021	7	ns	ns	ns
Decorin	Dcn	Male	1.76	0.14	8	1.74	0.12	8			
		Female	1.56	0.064	7	1.53	0.057	7	ns	ns	ns
Endothelin 1	Edn1	Male	0.0068	0.0003	7	0.00716	0.00056	8			
		Female	0.0066	0.00037	7	0.00708	0.00028	7	ns	ns	ns
Epidermal growth factor	Egf	Male	0.0132	0.0012	8	0.0134	0.002	8			
		Female	0.00875	0.0015	8	0.014	0.0013	8	ns	ns	ns
Endoglin	Eng	Male	0.159	0.0034	8	0.175	0.0094	8			
		Female	0.157	0.0085	7	0.158	0.005	7	ns	ns	ns
Fas ligand (TNF superfamily, member 6)	Fasl	Male	0.0000747	0.000014	7	0.00009	0.000021	7			
		Female	0.000116	0.000032	7	0.0000643	0.000017	7	ns	ns	ns
Gremlin 1	Grem1	Male	0.0000251	0.0000045	7	0.0000182	0.0000029	7			
		Female	0.0000185	0.0000025	7	0.0000185	0.000003	7	ns	ns	ns
Hepatocyte growth factor	Hgf	Male	0.00137	0.000081	8	0.00133	0.00011	8			
		Female	0.00142	0.00013	7	0.00135	0.0001	7	ns	ns	ns
Interferon gamma	Ifng	Male	0.000123	0.000045	8	0.00011	0.000026	8			
		Female	0.00014	0.000054	8	0.000161	0.000036	7	ns	ns	ns
Interleukin 10	Il10	Male	0.000174	0.000048	8	0.000236	0.000056	8			
		Female	0.000171	0.00004	7	0.000205	0.000045	8	ns	ns	ns
Interleukin 13	Il13	Male	0.000112	0.000039	8	0.00011	0.000022	8			
		Female	0.000103	0.000027	8	0.0000806	0.000015	8	ns	ns	ns
Interleukin 13 receptor, alpha 2	Il13ra2	Male	0.000052	0.0000083	8	0.0000631	0.0000083	8			
		Female	0.000055	0.000015	8	0.0000376	0.0000064	7	ns	ns	ns
Interleukin 1 alpha	Il1a	Male	0.000554	0.000042	8	0.00053	0.00011	8			
		Female	0.00058	0.000074	7	0.000719	0.0001	8	ns	ns	ns
Interleukin 1 beta	Il1b	Male	0.00379	0.00065	7	0.0027	0.00049	7			
		Female	0.00332	0.00027	8	0.00329	0.00068	8	ns	ns	ns
Interleukin 4	Il4	Male	0.000194	0.000041	8	0.000309	0.000081	8			
		Female	0.000234	0.00003	8	0.000187	0.000042	8	ns	ns	ns
Interleukin 5	Il5	Male	0.000322	0.000082	8	0.000323	0.000032	8			
		Female	0.000353	0.000071	8	0.000482	0.000077	7	ns	ns	ns
Integrin linked kinase	Ilk	Male	0.151	0.0058	7	0.145	0.0033	7			
		Female	0.142	0.006	7	0.143	0.0026	7	ns	ns	ns
Inhibin beta E	Inhbe	Male	0.0000214	0.0000032	7	0.000014	0.0000013	8			
		Female	0.0000208	0.0000028	7	0.0000249	0.0000051	8	ns	ns	ns
Integrin alpha 1	Itga1	Male	0.0797	0.0043	8	0.0819	0.0048	8			
		Female	0.0763	0.0028	7	0.0748	0.003	7	ns	ns	ns
Integrin alpha 2	Itga2	Male	0.00114	0.00014	7	0.00125	0.000081	7			
		Female	0.00149	0.00007	8	0.00139	0.000093	8	ns	*	ns
Integrin alpha 3	Itga3	Male	0.00739	0.00054	8	0.00758	0.00065	8			
		Female	0.00836	0.00076	8	0.00753	0.00034	7	ns	ns	ns

Integrin alpha V	Itgav	Male	0.0574	0.0029	8	0.0588	0.0024	8			
		Female	0.0579	0.0016	7	0.0641	0.004	8	ns	ns	ns
Integrin beta 1 (fibronectin receptor beta)	Itgb1	Male	0.679	0.023	8	0.615	0.032	8			
		Female	0.643	0.028	8	0.636	0.053	8	ns	ns	ns
Integrin beta 3	Itgb3	Male	0.00357	0.0006	7	0.00336	0.00028	7			
		Female	0.00352	0.00035	8	0.00339	0.00027	8	ns	ns	ns
Integrin beta 5	Itgb5	Male	0.0857	0.0037	8	0.0903	0.001	8			
		Female	0.0808	0.0034	8	0.0982	0.0073	8	ns	ns	*
Integrin beta 6	Itgb6	Male	0.0676	0.0041	8	0.0759	0.0065	8			
		Female	0.0782	0.0056	8	0.0912	0.0098	8	ns	ns	ns
Integrin beta 8	Itgb8	Male	0.00315	0.00044	8	0.00338	0.00033	8			
		Female	0.00321	0.000097	7	0.00333	0.00022	8	ns	ns	ns
Jun oncogene	Jun	Male	0.127	0.0035	8	0.11	0.0068	8			
		Female	0.112	0.0092	8	0.117	0.01	8	ns	ns	ns
Lysyl oxidase	Lox	Male	0.00679	0.0004	7	0.00518	0.00045	7			
		Female	0.00528	0.0004	8	0.00483	0.00046	8	ns	*	*
Latent transforming growth factor beta binding	Ltbp1	Male	0.0974	0.0068	8	0.104	0.0083	8			
protein 1		Female	0.0973	0.0024	7	0.102	0.0057	7	ns	ns	ns
Matrix metallopeptidase 13	MMP-13	Male	0.00234	0.00018	8	0.00198	0.00018	8			
		Female	0.00235	0.00016	7	0.00183	0.00019	7	ns	ns	*
Matrix metallopeptidase 14 (membrane-	Mmp14	Male	0.0143	0.00082	8	0.019	0.0022	8			
inserted)		Female	0.0177	0.0012	8	0.019	0.0022	8	ns	ns	ns
Matrix metallopeptidase 1a (interstitial	Mmp1a	Male	1.19	0.086	7	1.18	0.086	8			
collagenase)		Female	1.33	0.13	8	1.01	0.095	7	ns	ns	ns
Matrix metallopeptidase 2	MMP-2	Male	0.0469	0.002	8	0.0539	0.0013	7			
		Female	0.0526	0.0051	8	0.0714	0.0065	8	ns	*	**
Matrix metallopeptidase 3	Mmp3	Male	0.00744	0.00093	8	0.00777	0.00088	8			
		Female	0.00411	0.00036	8	0.00423	0.00033	7	ns	****	ns
Matrix metallopeptidase 8	Mmp8	Male	0.000118	0.000029	8	0.000112	0.000035	7			
		Female	0.000125	0.000037	8	0.0000481	0.000016	8	ns	ns	ns
Matrix metallopeptidase 9	MMP-9	Male	0.00167	0.00027	8	0.00259	0.00019	7			
		Female	0.00176	0.00035	7	0.00272	0.00024	8	ns	ns	**
Myelocytomatosis oncogene	Myc	Male	0.00446	0.00036	7	0.00494	0.00057	7			
		Female	0.00522	0.00033	8	0.00576	0.00052	8	ns	ns	ns
Nuclear factor of kappa light polypeptide gene	Nfkb1	Male	0.0559	0.0017	8	0.0558	0.0026	8			
enhancer in B-cells 1, p105		Female	0.053	0.003	8	0.056	0.0018	7	ns	ns	ns
Platelet derived growth factor, alpha	Pdgfa	Male	0.0127	0.00066	7	0.0145	0.00041	7			
		Female	0.0134	0.0011	8	0.0166	0.0016	8	ns	ns	*
Platelet derived growth factor, B polypeptide	Pdgfb	Male	0.0629	0.0045	8	0.0616	0.0035	8			
		Female	0.0587	0.0046	8	0.052	0.0026	8	ns	ns	ns
Plasminogen activator, tissue	Plat	Male	0.034	0.0019	8	0.0355	0.0017	8			
		Female	0.0395	0.0018	8	0.0351	0.0013	7	ns	ns	ns
Plasminogen activator, urokinase	Plau	Male	0.00523	0.00027	8	0.00663	0.00081	8			
		Female	0.00384	0.00021	7	0.00516	0.00034	8	ns	**	**
Plasminogen	Plg	Male	0.0000793	0.00002	7	0.0000946	0.000023	8			
		Female	0.00113	0.00069	7	0.000197	0.000042	6	ns	ns	ns
Serine (or cysteine) peptidase inhibitor, clade	Serpina1a	Male	0.00149	0.00085	7	0.000167	0.000079	6			
A, member 1a		Female	0.0000332	0.000015	7	0.0000134	0.0000013	7	ns	ns	ns
Serine (or cysteine) peptidase inhibitor, clade E,	Serpine1	Male	0.0311	0.0068	8	0.0231	0.0032	8			
member 1		Female	0.0229	0.0028	8	0.0223	0.0013	7	ns	ns	ns
Serine (or cysteine) peptidase inhibitor, clade	Serpinh1	Male	0.272	0.016	8	0.293	0.022	8			
H, member 1		Female	0.235	0.017	8	0.269	0.015	8	ns	ns	ns
MAD homolog 2 (Drosophila)	Smad2	Male	0.0325	0.00074	7	0.0328	0.0018	8			
		Female	0.0324	0.0012	8	0.0309	0.00087	7	ns	ns	ns
MAD homolog 3 (Drosophila)	Smad3	Male	0.0164	0.0009	8	0.018	0.0014	8			
		Female	0.0169	0.00044	7	0.0196	0.0013	8	ns	ns	ns
MAD homolog 4 (Drosophila)	Smad4	Male	0.187	0.0058	7	0.185	0.014	8			
		Female	0.176	0.0087	8	0.165	0.0033	7	ns	ns	ns
MAD homolog 6 (Drosophila)	Smad6	Male	0.0127	0.00095	8	0.0162	0.002	8			
		Female	0.0115	0.0011	8	0.012	0.00063	7	ns	ns	ns
MAD homolog 7 (Drosophila)	Smad7	Male	0.0153	0.001	7	0.0164	0.0026	7			
		Female	0.00996	0.00081	7	0.0126	0.0012	7	ns	**	ns
Snail homolog 1 (Drosophila)	Snai1	Male	0.000321	0.000026	7	0.000388	0.00004	8			
		Female	0.00036	0.000052	7	0.000421	0.000073	8	ns	ns	ns
Trans-acting transcription factor 1	Sp1	Male	0.0441	0.0025	8	0.042	0.0017	8			
		Female	0.0435	0.0014	8	0.0425	0.00098	7	ns	ns	ns
Signal transducer and activator of transcription	Stat1	Male	0.0643	0.0036	8	0.0677	0.0026	7			
1		Female	0.0828	0.0063	8	0.074	0.0043	8	ns	*	ns
Signal transducer and activator of transcription	Stat6	Male	0.00465	0.00042	8	0.00484	0.00039	8			
6		Female	0.00372	0.00035	8	0.00417	0.00048	8	ns	ns	ns
Transforming growth factor, beta 1	TGF-β1	Male	0.0785	0.0052	8	0.0782	0.0026	8			
		Female	0.0714	0.0035	7	0.0688	0.0032	8	ns	*	ns
Transforming growth factor, beta 2	Tgfb2	Male	0.0139	0.0011	7	0.0175	0.0013	8			
		Female	0.0158	0.00098	8	0.0182	0.0012	8	ns	ns	*
Transforming growth factor, beta 3	Tgfb3	Male	0.0232	0.0014	8	0.0255	0.0012	8			
		Female	0.0214	0.0015	8	0.0233	0.0015	8	ns	ns	ns
Transforming growth factor, beta receptor I	Tgfbr1	Male	0.0186	0.00097	8	0.0179	0.00063	8			
		Female	0.0154	0.00082	8	0.0155	0.00074	8	ns	**	ns

Transforming growth factor, beta receptor II	Tgfbr2										
		Male	0.071	0.0028	8	0.0681	0.0021	8			
		Female	0.0637	0.0054	8	0.0668	0.0049	8	ns	ns	ns
TGFβ-induced factor homeobox 1	Tgif1	Male	0.00209	0.00016	8	0.00245	0.00024	8			
		Female	0.00229	0.00012	8	0.00265	0.00022	8	ns	ns	ns
Thrombospondin 1	Thbs1	Male	0.0464	0.011	8	0.0327	0.0052	8			
		Female	0.0193	0.0023	8	0.0147	0.0016	8	ns	***	ns
Thrombospondin 2	Thbs2	Male	0.0254	0.0025	8	0.0197	0.0017	8			
		Female	0.0234	0.0013	8	0.0239	0.0013	8	ns	ns	ns
Tissue inhibitor of metalloproteinase 1	TIMP1	Male	0.00267	0.00041	8	0.00172	0.00051	7			
		Female	0.00312	0.00036	8	0.0022	0.00053	8	ns	ns	*
Tissue inhibitor of metalloproteinase 2	TIMP2	Male	0.135	0.0056	8	0.126	0.0024	7			
		Female	0.122	0.0026	7	0.146	0.0058	8	**	ns	ns
Tissue inhibitor of metalloproteinase 3	Timp3	Male	0.177	0.023	8	0.137	0.0046	7			
		Female	0.144	0.0093	8	0.149	0.004	8	ns	ns	ns
Tissue inhibitor of metalloproteinase 4	Timp4	Male	0.102	0.014	7	0.0813	0.0086	7			
		Female	0.0715	0.0066	8	0.0675	0.0071	7	ns	*	ns
Tumor necrosis factor	Tnf	Male	0.00149	0.00018	8	0.0014	0.00017	8			
		Female	0.00171	0.00046	8	0.000904	0.00014	8	ns	ns	ns
Vascular endothelial growth factor A	Vegfa	Male	0.481	0.02	8	0.518	0.02	8			
		Female	0.452	0.017	7	0.463	0.024	8	ns	ns	ns
